# Nanopore Sequencing Reveals Global Transcriptome Signatures of Mitochondrial and Ribosomal Gene Expressions in Various Human Cancer Stem-like Cell Populations

**DOI:** 10.3390/cancers13051136

**Published:** 2021-03-06

**Authors:** Kaya E. Witte, Oliver Hertel, Beatrice A. Windmöller, Laureen P. Helweg, Anna L. Höving, Cornelius Knabbe, Tobias Busche, Johannes F. W. Greiner, Jörn Kalinowski, Thomas Noll, Fritz Mertzlufft, Morris Beshay, Jesco Pfitzenmaier, Barbara Kaltschmidt, Christian Kaltschmidt, Constanze Banz-Jansen, Matthias Simon

**Affiliations:** 1 Department of Cell Biology, Faculty of Biology, University of Bielefeld, Universitätsstrasse 25, 33699 Bielefeld, Germany; Beatrice.windmoeller@uni-bielefeld.de (B.A.W.); l.helweg@uni-bielefeld.de (L.P.H.); Anna.hoeving@uni-bielefeld.de (A.L.H.); Johannes.greiner@uni-bielefeld.de (J.F.W.G.); Barbara.kaltschmidt@uni-bielefeld.de (B.K.); C.Kaltschmidt@uni-bielefeld.de (C.K.); 2 Forschungsverbund BioMedizin Bielefeld, OWL (FBMB e.V.), Maraweg 21, 33699 Bielefeld, Germany; cknabbe@hdz-nrw.de (C.K.); fritz.mertzlufft@evkb.de (F.M.); Morris.Beshay@evkb.de (M.B.); Jesco.Pfitzenmaier@evkb.de (J.P.); Constanze.Banz-Jansen@evkb.de (C.B.-J.); Matthias.Simon@evkb.de (M.S.); 3 Department of Cell Culture Technology, Faculty of Technology, University of Bielefeld, Universitätsstrasse 25, 33699 Bielefeld, Germany; oliver.hertel@uni-bielefeld.de (O.H.); Thomas.Noll@uni-bielefeld.de (T.N.); 4 Center for Biotechnology-CeBiTec, University of Bielefeld, Universitätsstrasse 27, 33699 Bielefeld, Germany; tbusche@cebitec.uni-bielefeld.de (T.B.); joern@cebitec.uni-bielefeld.de (J.K.); 5 Heart and Diabetes Centre NRW, Institute for Laboratory and Transfusion Medicine, Ruhr-University Bochum, 32545 Bad Oeynhausen, Germany; 6 Scientific Director of the Protestant Hospital of Bethel Foundation, University Medical School OWL at Bielefeld, Bielefeld University, Campus Bielefeld-Bethel, Maraweg 21, 33699 Bielefeld, Germany; 7 Department for Thoracic Surgery and Pneumology, Protestant Hospital of Bethel Foundation, University Medical School OWL at Bielefeld, Bielefeld University, Campus Bielefeld-Bethel, Burgsteig 13, 33699 Bielefeld, Germany; 8 Department of Urology and Center for Computer-Assisted and Robotic Urology, Protestant Hospital of Bethel Foundation, University Medical School OWL at Bielefeld, Bielefeld University, Campus Bielefeld-Bethel, Burgsteig 13, 33699 Bielefeld, Germany; 9 Molecular Neurobiology, Faculty of Biology, Bielefeld University, Universitätsstrasse 25, 33699 Bielefeld, Germany; 10 Department of Gynecology and Obstetrics, and Perinatal Center, Protestant Hospital of Bethel Foundation, University Medical School OWL at Bielefeld, Bielefeld University, Campus Bielefeld-Bethel, Burgsteig 13, 33699 Bielefeld, Germany; 11 Department of Neurosurgery and Epilepsy Surgery, Protestant Hospital of Bethel Foundation, University Medical School OWL at Bielefeld, Bielefeld University, Campus Bielefeld-Bethel, Burgsteig 13, 33699 Bielefeld, Germany

**Keywords:** cancer stem cells, endometrioid carcinoma, glioblastoma multiforme, lung adenocarcinoma, prostate adenocarcinoma, nanopore sequencing, mitochondrion, ribosome

## Abstract

**Simple Summary:**

Cancer is the leading cause of death in the industrialized world. In particular, so-called cancer stem cells (CSCs) play a crucial role in disease progression, as they are known to contribute to tumor growth and metastasis. Thus, CSCs are heavily investigated in a broad range of cancers. Nevertheless, global transcriptomic profiling of CSC populations derived from different tumor types is rare. We established three CSC populations from tumors in the uterus, brain, lung, and prostate and assessed their global transcriptomes using nanopore full-length cDNA sequencing, a new technique to assess insights into global gene profile. We observed common expression in all CSCs for distinct genes encoding proteins for organelles, such as ribosomes, mitochondria, and proteasomes. Additionally, we detected high expressions of inflammation- and immunity-related genes. Conclusively, we observed high similarities between all CSCs independent of their tumor of origin, which may build the basis for identifying novel therapeutic strategies targeting CSCs.

**Abstract:**

Cancer stem cells (CSCs) are crucial mediators of tumor growth, metastasis, therapy resistance, and recurrence in a broad variety of human cancers. Although their biology is increasingly investigated within the distinct types of cancer, direct comparisons of CSCs from different tumor types allowing comprehensive mechanistic insights are rarely assessed. In the present study, we isolated CSCs from endometrioid carcinomas, glioblastoma multiforme as well as adenocarcinomas of lung and prostate and assessed their global transcriptomes using full-length cDNA nanopore sequencing. Despite the expression of common CSC markers, principal component analysis showed a distinct separation of the CSC populations into three clusters independent of the specific type of tumor. However, GO-term and KEGG pathway enrichment analysis revealed upregulated genes related to ribosomal biosynthesis, the mitochondrion, oxidative phosphorylation, and glycolytic pathways, as well as the proteasome, suggesting a great extent of metabolic flexibility in CSCs. Interestingly, the GO term “NF-kB binding” was likewise found to be elevated in all investigated CSC populations. In summary, we here provide evidence for high global transcriptional similarities between CSCs from various tumors, which particularly share upregulated gene expression associated with mitochondrial and ribosomal activity. Our findings may build the basis for identifying novel therapeutic strategies targeting CSCs.

## 1. Introduction

Cancer stem cells (CSCs) are increasingly noticed to initiate tumor growth and to drive metastasis and tumor recurrence in a broad range of human cancers (reviewed in [1]). Within the highly heterogeneous tumor cell mass, CSCs represent only a small subpopulation [2] (reviewed in [3]), but possess stem-like properties like self-renewal, asymmetric division and multi-lineage differentiation [4,5,6,7] (reviewed in [1]). Moreover, CSCs remain hidden in the body of the patients until their reactivation by various stimuli leads to the regeneration of the tumor or to the formation of metastasis [8]. These characteristics facilitate the role of CSCs as major drivers of tumor formation and progression. From a therapeutic point of view, their quiescent-like state makes CSCs highly resistant to chemotherapeutic agents, while the low expression of major histocompatibility class I molecules enables the escape from immune surveillance by cytotoxic T-cells [9,10,11]. To gain a deeper understanding of CSC biology and potential treatment options, global transcriptional profiling has become a state-of-the-art tool during the recent years [12]. However, direct comparisons of CSCs from different tumor types are rarely assessed, although these may allow the identification of comprehensive mechanisms present in CSCs independent to the type of the tumor. In the present study, we isolated CSCs from endometrioid carcinomas, glioblastoma multiforme, as well as adenocarcinomas of lung and prostate, and assessed their global transcriptomes by nanopore RNA sequencing (RNA-Seq) to identify such potential common regulators and mechanisms.

Endometrial cancer is one of the most common sex-specific malignant diseases in women worldwide. Annually, about 320,000 women are diagnosed with endometrial cancer. Especially in high-income countries, the incidence of endometrial cancer is high, at 5.9% [13]. Major risk factors are obesity, physical inactivity, and elevated estrogen levels in postmenopausal women [14,15]. In Europe and North America, endometrial cancer is the most frequent cancer of the female genital tract. However, it is mainly presented with postmenopausal bleeding and therefore in most cases diagnosed at an early stage. Nevertheless, it is more and more emphasized that a small subpopulation of tumor stem-like cells with clonogenic, self-renewing, differentiating and tumorigenic properties are responsible for the production of endometrial carcinoma cells [4]. Additionally, endometrial CSCs seem to play a role in chemoresistance of endometrial carcinomas, as increased expression of CSC markers were shown to enrich resistance to cisplatin, placlitaxel and doxorubicin [16].

Being the most common primary brain tumor, glioblastoma multiforme (GBM) possesses a high cellular heterogeneity and aggressiveness accompanied by an extensive invasiveness and inevitable recurrence, resulting in an average survival time of less than 15 months [6,17,18,19]. As a description of the cellular composition, GBM tumors contain a relatively rare glioblastoma stem-like cell (GSC) population, which is able to self-renew and repopulate the whole tumor building [20]. GSCs can be found within the tumor infiltrating zone and therefore contribute prominently to a subsequent tumor recurrence [21]. On the contrary, differentiated GBM cells are considered as the main contributor to the tumor mass development [20,22,23,24,25,26]. In a therapeutic context, Happold and coworkers analyzed GSCs and described NF-κB RELA as a positive regulator of O6-methylguanine-DNA methyltransferase (MGMT) [27]. Of note, *MGMT* promoter hypermethylation has proven an important predictive biomarker for benefit from alkylating chemotherapy as well as a powerful prognostic factor in gliomas [28,29].

Lung cancer is the leading cause of cancer-related deaths worldwide. According to its histological differentiation, it is classified into small-cell lung cancer and non-small cell lung cancer (NSCLC), which is the most frequent form with an incidence of about 80% [30]. In the last few years, target therapy or immune therapy has been gaining popularity. Nevertheless, the overall prognosis of NSCLC is bad, with a five-year survival rate of only 15% [31]. A meta-analysis evaluating the effect of CSC markers on the clinicopathological characteristics of lung cancer revealed a significant association with poor differentiation and metastasis [32]. Accordingly, CSCs were reported to be responsible for therapy resistance and tumor growth as well as metastasis in lung cancer [5]. Moreover, lung cancer stem-like cells (LCSCs) were shown to be regulated by NF-κB [33], as already mentioned for GSCs.

Prostate cancer (PCa) is the most common sex-specific cancer within industrialized countries as well as the second leading cause of cancer deaths (10%) in men [34]. PCas are described as having an epithelial origin and an almost exclusive occurrence as an acinar adenocarcinoma [35]. PCa mainly occurs in the elderly, from the age of at least 65 years, and can be detected using prostate-specific antigen-testing, enabling early stage detection [36]. The overall mortality is only reduced by performing a radically prostatectomy [37]. Relating to further therapeutic options, luminal epithelial stem cells could be revealed as the origin of PCa via lineage tracing in mice [38]. In context with CSCs, prostate tumor spheres, originating from prostate cancer stem-like cells (PCSCs), were also shown to express a constitutive NF-κB signaling and inherent increased IL-6 levels [39].

Here, we established cultures of CSCs and analyzed three of each cancer type via RNA-Seq on global transcriptome level. We used the recently developed nanopore sequencing technology (reviewed in [40]) and a protocol for generating full length cDNA to identify common regulators and mechanisms present in CSCs independent to the tumor origin.

## 2. Materials and Methods

### 2.1. Cancer Stem-like Cell Population Establishment and Cultivation

Cancer tissue samples used to isolate CSCs were obtained during surgical resection and were kindly provided by the Forschungsverbund BioMedizin Bielefeld/OWL (FBMB e.V.) at the Protestant Hospital of Bethel Foundation (Bielefeld, Germany) after assuring routine histopathological analysis. Primary tumor samples were collected from each tumor type, including three endometrioid carcinomas, three glioblastomas, and three adenocarcinomas of the lung and prostate, respectively. Informed consent according to local and international guidelines was signed by all patients and further experimental procedures were ethically approved (Ethics committee Münster, Germany, 2017-522-f-S).

Samples were transferred into ice-cold Dulbecco’s phosphate buffered saline (PBS; Sigma Aldrich, Munich, Germany) and for further processing transported to the University of Bielefeld. Tumor tissue was mechanically disintegrated followed by enzymatic digestion with collagenase for 2 h at 37 °C as described previously [41,42]. The minced tissue was cultured on gelatin (bovine skin-derived, type B; Sigma Aldrich)-coated culture dishes in CSC-selective medium composed of Dulbecco’s modified Eagle’s medium/Ham’s F-12 (Sigma Aldrich) with the addition of 2 mM L-glutamine (Sigma Aldrich), penicillin/streptomycin (100 μg/mL; Sigma Aldrich), epidermal growth factor (EGF; 20 ng/mL; MiltenyiBiotec, Bergisch Gladbach, Germany), basic fibroblast growth factor-2 (bFGF-2; 40 ng/mL; Miltenyi Biotec), B27 supplement (Gibco, Thermo Fisher Scientific, Bremen, Germany) and 10% FCS (Sigma Aldrich). Enrichment of CSCs was achieved via serial trypsin treatment, as described by Walia et al. and Morata-Tarifa et al. [43,44]. Briefly, cells isolated by explant culture (passage 0) were washed with PBS and subsequently treated for 5 min with trypsin (Sigma Aldrich). Detached cells were transferred into a new gelatin pre-coated culture dish. Trypsinization and transfer of the cells were repeated every 48 to 72 h, at least for three cycles to assure stem-like characteristics in adherently grown and fibroblast-shaped cancer cells. For cultivation of free-floating spheres, CSC populations from endometrioid carcinoma and glioblastoma multiforme were cultured without the addition of serum for several days in regular growth medium supplemented with 4 µg/mL heparin (Sigma Aldrich).

### 2.2. Immunocytochemistry

For immunocytochemical staining of adherent CSC populations, cells were seeded at the top of sterilized coverslips with 15,000-30,000 cells per 4 cm^2^ in 24-well plates with 0.5 mL growth medium and cultured initially for 48 to 72 h until cells reached 70–80% confluency. CSCs were fixed for 10 min with 4% para-formaldehyde in PBS. Blocking of free binding sides and permeabilization were performed with PBT solution, including 0.02% Triton-X-100 (Sigma Aldrich) and 5% goat serum (DIANOVA, Hamburg Germany) in PBS for 30 min. Next, three washing steps with PBS were performed as well as an incubation with primary antibodies for 1 h at room temperature (RT). Used antibodies for this study: anti-CD44 (1:400; 156-3C11; Cell Signaling, Frankfurt am Main, Germany), anti-CD133 (1:100; NB120-16518; NovusBio, Bio-Techne, Wiesbaden-Nordenstadt, Germany), anti-Nestin (1:200; MAB5326; Merck) and anti-MYC (10 μg/mL; Y69; Abcam, Cambridge, UK). After further washing steps, secondary fluorochrome-conjugated antibodies (Alexa Fluor 555 and -488 dyes; 1:300; goat anti-mouse and goat anti-rabbit; Life Technologies) were applied for 1 h at RT, protected from light. Nuclear staining were performed by using 4′,6-diamidino-2-phenylindole (DAPI; 1 µg/mL; Sigma Aldrich) for 10 min. Before CSC populations were embedded in Mowiol 4-88 (Carl Roth GmbH, Karlsruhe, Germany) upside down on the top of microscope slides, another washing step was performed. For fluorescence imaging, a confocal laser-scanning microscope (LSM 780; Carl Zeiss, Jena, Germany) was used.

For immunostaining of CSC spheres, free-floating cultured cells were fixed in 4% para-formaldehyde for 2 h, were further embedded in paraffin and sectionalized in 4 µm sections. Resulting slices were deparaffinized and rehydrated with xylol (Sigma Aldrich) as well as via ethanol in different steps. After reconditioning of the epitope with citrate buffer (pH 6), slices were washed in PBS and blocking of free binding sides were performed via incubation with 0.02% Triton-X 100, 10% appropriative serum and 1% bovine serum albumin (Sigma Aldrich) also in PBS for at least 2 h at RT. Next, incubation with primary antibodies was performed over night at 4 °C by using: anti-CD44 (1:50), anti-CD133 (1:100) and anti-MYC (10 µg/mL). Slices were washed three times with PBS and incubated for 1 h at RT with the Alexa Fluor 555 and −488 secondary fluorochrome-conjugated antibodies (1:300). Nuclear staining by using DAPI (1 µg/mL) as well as fluorescence imaging were processed equally to immunocytochemistry of adherently cultured CSCs.

### 2.3. RNA Isolation and Sequencing

RNA from 1 × 10^6^ cultured CSCs of each population and cancer type were isolated by using the NucleoSpin^®^ RNA Plus kit (Macherey-Nagel, Düren, Germany) according to manufacturer’s guidelines. Quality and concentration of isolated RNAs were assessed via nanodrop ultraviolet spectrophotometry. Total RNA samples with RNA Integrity Numbers (RIN) > 9.5 were used to convert full-length RNA molecules that are both capped and polyadenylated to cDNA using the TeloPrime Full-Length cDNA Amplification Kit V2 (Lexogen, Vienna, Austria). Amplified full length cDNAs were then used to prepare Oxford Nanopore Technologies (ONT) compatible libraries using the Ligation Sequencing Kit LSK109 with the Native Barcoding Kit NBD104 (ONT, Oxford, UK), which were run on three R9.4 flowcells on the ONT system GridION. Base calling and demultiplexing were performed using Guppy v3.1.5.

### 2.4. Preprocessing and Genome Alignment

Fastq files containing reads that passed the quality filtering were concatenated according to their barcodes from each flowcell. Since the three flowcells showed high correlation on gene count level, technical triplicates were merged accordingly. Sequencing adapters were trimmed using porechop v0.2.4 [45]. Trimming was checked using FastQC v0.11.9 [46]. Trimmed reads were aligned to the human RefSeq genome GRCh38.p13 [47,48] using minimap 2 [49] with the arguments -ax splice -p 0.99. Alignment files were converted to bam format using samtools v1.10.2 [50]. The bam files were quality checked using AlignQC v2.0.5 [51] samtools v1.10.2 [50]. As the alignments showed up to 15% trans-chimeric reads, which most likely resulted from the library preparation, those reads were removed from the alignments. Therefore, the chimera.bed file from the AlignQC output were converted to exclusion lists. Bam files were sorted using samtools v1.10.2 [50] and filtered using the FilterSamReads module of Picard Toolkit (http://broadinstitute.github.io/picard/ (accessed on 6 March 2020)) with the exclusion lists and the arguments --FILTER excludeReadList --SORT_ORDER. Mismatches and small indels were corrected using TranscriptClean [52] with the human RefSeq genome GRCh38.p13 [47]. These high-quality alignments were used as input for the estimation of gene abundances.

### 2.5. Gene Abundance Estimation and Enrichment Analysis

Mapped reads were assigned to genes and counted by the featureCounts module of the R/Bioconductor [53] package Rsubread v2.0.1 [54] with the arguments countMultiMappings = TRUE, fraction = TRUE, isLongRead = TRUE. The gtf file of the human RefSeq genome GRCh38.p13 [47] was used as external annotation. From genes with multiple integrations but the same exon structure, only one was retained, because they shared the same multimapping reads. These raw counts were grouped by the CSC populations and preprocessed using the R/Bioconductor package edgeR v3.28.1. [55,56]. Lowly expressed genes were filtered using the filterByExpr function with default arguments. Normalization factors were calculated using the trimmed mean of M-values method [57]. Differentially expressed genes (DEGs) were identified by an ANOVA-like testing using the generalized linear models with the quasi-likelihood F-test (glmQLFTest). The threshold of DEGs was set as *p* value < 0.05. Principle component analysis (PCA) was conducted using the R/Bioconductor package PCAtools v1.2.0 [58]. Heatmaps were created using the heatmap.2 function of the R package gplots v3.1.1 [59]. Correlation analysis was conducted using the R package Hmisc package [60]. Functional enrichment of GO-terms of genes expressed in all 12 CSC populations were calculated using the PANTHER classification system [61], while the DAVID database [62,63] served for calculating functional enriched KEGG pathways. Significantly (modified Fisher Exact p-value; *p* < 0.05) enriched GO-terms and pathways were visualized with Prism software (GraphPad Software, San Diego, CA, USA).

## 3. Results

### 3.1. Correlative Analysis of Characteristic Markers in Cancer Stem-like Cells from Endometrioid Carcinomas, Glioblastoma Multiforme, Lung- and Prostate Adenocarcinomas

In this study, 12 different CSC populations from four different carcinoma types were established. Carcinoma types included three endometrial carcinomas, three glioblastomas as well as three adenocarcinomas of the lung and the prostate, respectively. All female donors of the endometrial cancer stem-like cell populations (ECSCs) suffered from endometrioid carcinomas of the corpus uteri ranging WHO grade I-II and were aged between 72 and 86 years. Donors of GSCs were two males (42 and 69 years old) and one female (60 years old), all revealing glioblastoma multiforme. Glioblastomas of the donors of GSCs_a and GSCs_c revealed no mutation for the isocitrate dehydrogenase (NADP(+)) 1 (*IDH1*), whereas in the glioblastoma of the donor of GSCs_b *IDH1* was mutated. Additionally, *MGMT* promoter methylation status differed between the three donors, as glioblastomas of the donors of GSCs_a and GSCs_b depicted *MGMT* promoter methylation in contrast to the glioblastoma of donor GSCs_c. LCSC populations were derived from three relatively young female patients (aged between 49 and 61 years) all diseased with adenocarcinomas. Analysis of clinically relevant mutations revealed an epidermal growth factor receptor (*EGFR*) mutation for the donor of LCSCs_a as well as tumor tissue of the donor of LCSCs_c showed mutations in the genes for the KRAS proto-oncogene (*KRAS*) and serine/threonine kinase 11 (*STK11*). The three PCSCs were isolated from male patients aged between 57 and 72 years all suffering from acinar adenocarcinomas with WHO grade II, III and V (Table 1).

**Figure 1:**
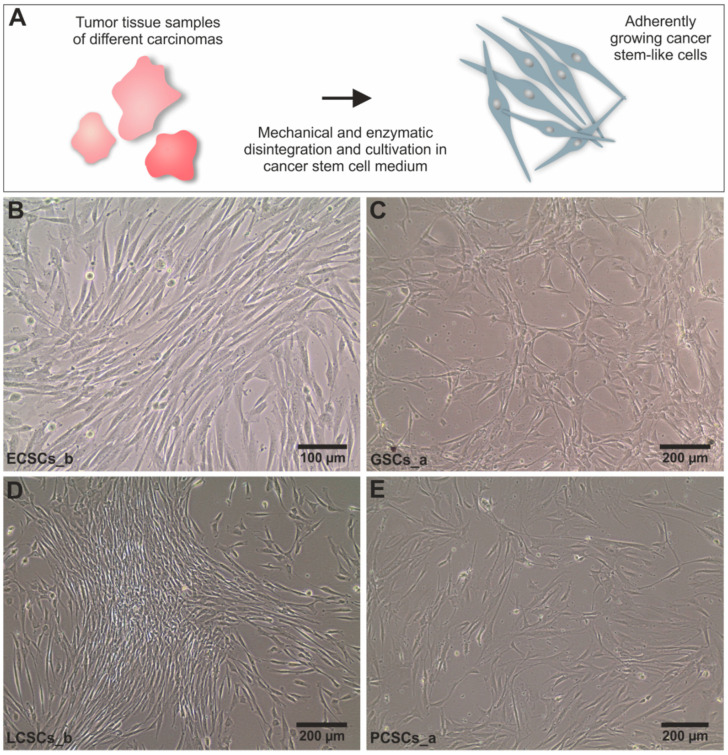
Isolation of cancer stem-like cell populations of four different types of carcinomas. (**A**) Schematic illustration of the isolation of cancer-stem-like cells out of primary tumor tissue. Representative pictures of adherently grown primary cancer stem-like cells derived from parental tumor tissue of (**B**) endometrial cancer stem-like cells b (ECSCs_b), (**C**) glioblastoma stem-like cells a (GSCs_a), (**D**) lung cancer stem-like cells b (LCSCs_b) and (**E**) prostate cancer stem-like cells a (PCSCs_a).

**Figure 2:**
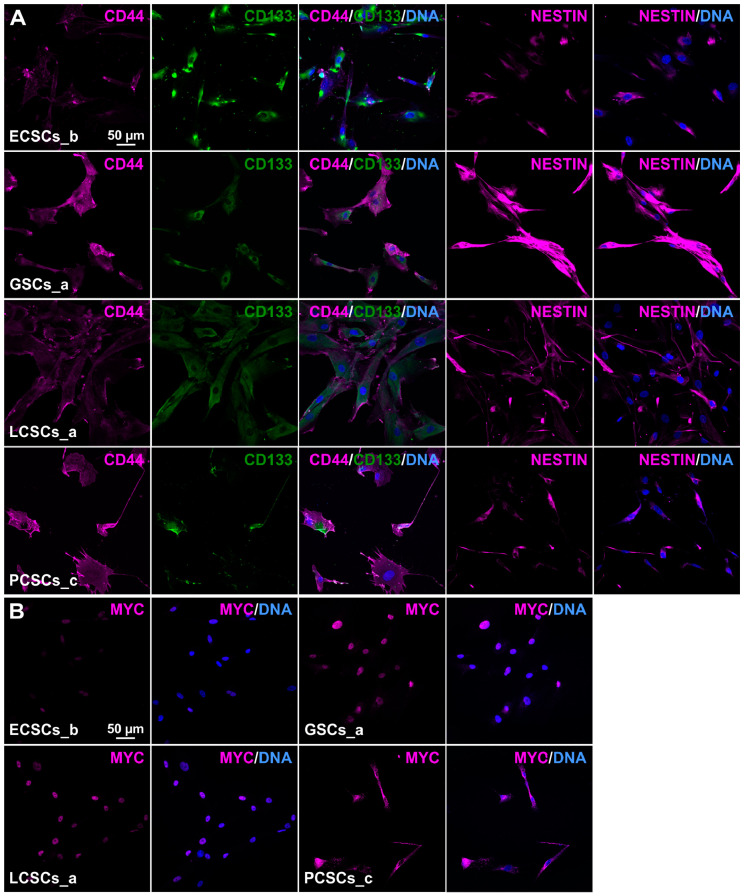
Immunocytochemistry of adherently grown cancer stem-like cell populations. (**A**) Representative immunostainings of co-expressed cancer stem cell (CSC) markers CD44/CD133 as well as Nestin, localized within the cytoplasm. (**B**) Exemplary depicted MYC expressions from one of each CSC population showed high frequencies of nuclear localizations of the analyzed protein. DAPI served in all cases for nuclear counterstaining.

All cancer populations were cultured as adherently growing cells within stem cell-selective media after passing serial trypsin treatment, for enrichment of the respective CSC-population. Here, we used chemically defined medium containing EGF and bFGF-2, in accordance with the isolation of colorectal cancer stem-like cells as well as of adult human stem cells from the nasal cavity and the heart auricle [42,64,65,66,67]. In contrast with the successful isolation of human stem cells from the nasal cavity as free-floating spheres [64,65], adoption of the reported isolation process for human CSCs from solid tumors resulted in an unsatisfactory low culture efficacy and low growth rates. Therefore, we utilized an alternative method to enrich primary isolated CSCs, namely via differential trypsinization [42,44]. In accordance with a range of previous studies, we obtained trypsin-sensitive CSC populations with low attachment capability by culturing with 10% FCS [42,43,44,66,67]. Representative images of one of the successfully isolated CSC populations derived from endometrioid carcinomas, glioblastomas, lung and prostate adenocarcinomas revealed similar morphology independently of the origin of parental tumor tissues (Figure 1B–E). In accordance with the observations by Walia et al. and Elble et al., cultured CSCs depicted a fibroblast-like and spindle-shaped morphology after selection with differential trypsin treatment [43]. To confirm the stem-like phenotype of isolated CSC populations, we determined the presence of characteristic CSC markers on protein level using immunocytochemistry. Notably, high levels of CD44, CD133, Nestin and MYC protein were detectable in adherently grown CSC populations from all tumor types as well as in spheres derived from ECSC_b and GSC_c (Figures S1 and S2 and Figure 2). Immunocytochemistry further revealed co-expressions of CD44 and CD133 as well as Nestin in isolated CSCs, independently to the tumor origin (Figure 2A and Figures S2 and S3). The proto-oncogene MYC was also detectable on protein level independent to the parental tumor tissue, particularly with a nuclear localization (Figure 2B and Figure S4). As an internal negative control and evaluation of the unique stem-like characteristics of isolated CSCs, human dermal fibroblasts (HDFs) were additionally immunocytochemically stained for CD44, CD133, Nestin and MYC. Here, HDFs showed no signs of CSC markers on protein level (Figure S5), emphasizing the CSC-like character of our isolated cell populations.

**Figure 3:**
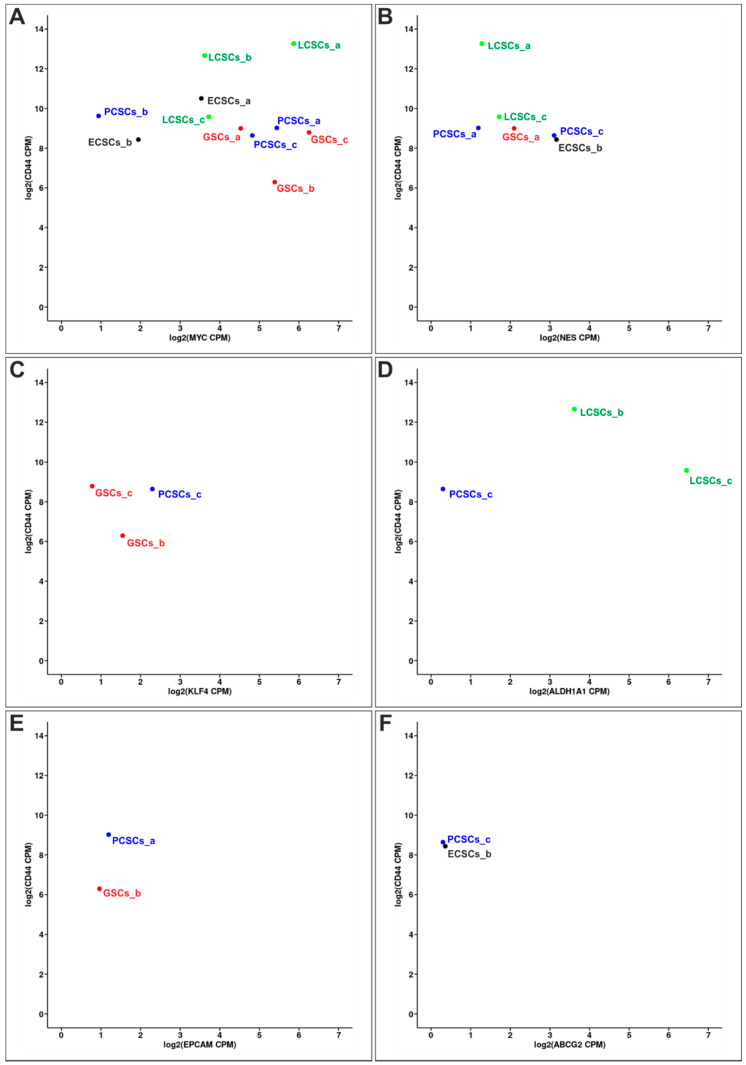
Correlated expression of CD44 with various other cancer stem cell (CSC) markers in different CSC populations. For each analysis, biological replicates depicted the mean of merged technical triplicates. (**A**) All CSC populations expressed CSC marker *CD44* and MYC proto-oncogene (*MYC*), except for endometrial cancer stem-like cell population c (ECSCs_c), which only expressed *CD44*. (**B**) CSC marker *Nestin* was expressed in lung cancer stem-like cells (LCSCs)_a and _c, prostate cancer stem-like cells (PCSCs)_a and _c as well as in glioblastoma stem-like cells (GSCs)_a and ECSCs_b. (**C**) Pluripotency marker Kruppel like factor 4 (*KLF4*) was expressed in GSCs_b, GSCs_c and in PCSCs_c as well as (**D**) aldehyde dehydrogenase 1 (*ALDH1*) could be detected in LCSCs_b, LCSCs_c and PCSCs_c. (**E**) Epithelial cell adhesion molecule (*EPCAM*) was only observed in PCSCs_a and GSCs_b. (**F**) Expression of the ATP binding cassette subfamily G member 2 (*ABCG2*) gene could only be detected in PCSCs_c and ECSCs_b.

For in-depth analysis of the gene expression of the 12 isolated CSC populations nanopore cDNA sequencing was used. Full-length cDNA sequencing was conducted using three R9.4 flowcells on the ONT system GridION. Only reads that passed the default quality criteria of the Guppy basecaller were further investigated. All sequencing runs yielded 10,627,235 reads (11.2 Gbp of sequence) with a mean read length of 1056 bp and a mean quality score of 21.3. Reads were aligned to the human genome (hg38) using minimap2 [49]. We observed a read alignment rate of 99.2% and an error rate of 7.3%, which was reduced to 0.69% using TranscriptClean [52] for correction. To check the reproducibility of gene counts between the technical triplicates, fastq files from the different flowcells were preprocessed, mapped, corrected, and quantified separately. Subsequent analysis showed strong correlation between respective flowcells (mean Spearman correlation coefficient ρ = 0.94). All datasets were processed similarly and gene expression of specific CSC markers was investigated firstly. CSC marker *CD44* was ubiquitously expressed in all 12 CSC populations. Analysis of the co-expression of *CD44* with further CSC markers revealed expression of MYC proto-oncogene (*MYC*) in all CSC populations, except for ECSCs_c. Here, especially LCSCs_a and GSCs_c showed high expression of *MYC* (Figure 3A). Further quantification depicted *Nestin* expression in LCSCs_a, _c, PCSCs_a, _c as well as in GSCs_a and ECSCs_b (Figure 3B). Pluripotency marker Kruppel-like factor 4 (*KLF4*) and aldehyde dehydrogenase 1 (*ALDH1*) were only expressed in three of 12 CSC populations each, as *KLF4* was only observable in GSCs_b, _c and PCSCs_c and *ALDH1* in LCSCs_b, _c as well as in PCSCs_c (Figure 3C,D). In CSC populations PCSCs_a and GSCs_b, expression of epithelial cell adhesion molecule (*EPCAM*) was detectable (Figure 3E). Additionally, PCSCs_c and ECSCs_b expressed ATP binding cassette subfamily G member 2 (*ABCG2*) (Figure 3F). On the contrary, we did not observe any expression of CSC markers Prominin-1 (*CD133*), SRY-box transcription factor 2 (*SOX2*), POU class 5 homeobox 1 (*OCT4*) and MYCN proto-oncogene in the here analyzed CSCs cultivated and sequenced as described above. In summary, all isolated CSC populations expressed the CSC marker *CD44*. Expression of further CSC markers was more heterogeneous, except for the expression level of *MYC*, which could be detected in 11 of 12 CSC populations. *Nestin*, *KLF4*, *ALDH1*, *EPCAM* as well as *ABCG2* expression was incongruous, with no clear relation to the four different parental tumor groups.

### 3.2. Global Gene Expression Analysis of Cancer Stem-like Cell Populations Reveals Distinct Clusters

**Figure 4:**
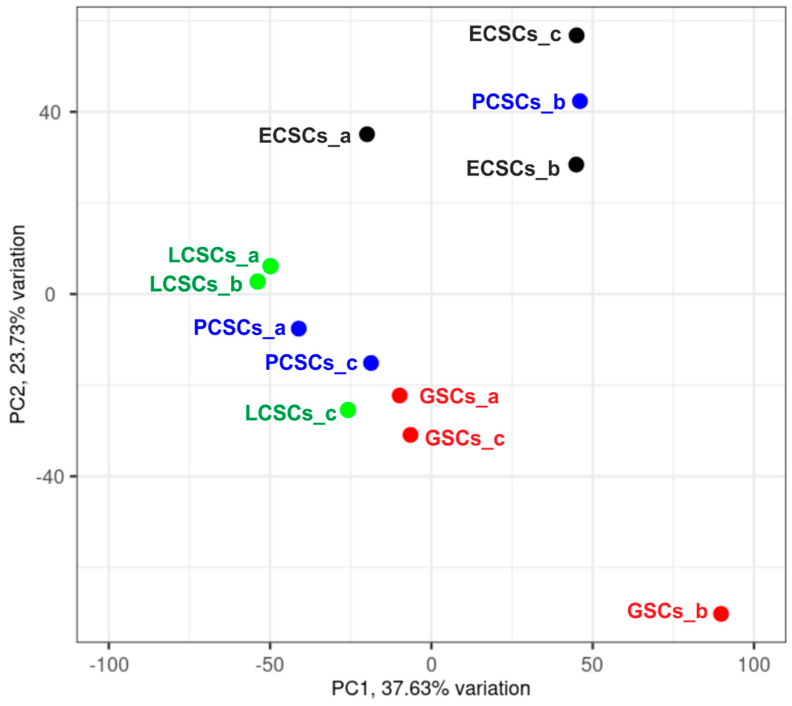
Comparison of gene expression profiles from different primary cancer stem-like cell (CSC) populations. Principal component analysis reveals two predominant clusters of CSCs, independently of the parental tumor types. Within the first cluster all three endometrial cancer stem-like cells (ECSCs) are present and one of the three prostate cancer stem-like cell (PCSCs) populations. The second cluster comprises the two remaining PCSCs, all three lung cancer-derived stem-like cell populations (LCSCs) as well as glioblastoma stem-like cells a (GSCs_a) and GSCs_c. The population of GSCs_b clustered independently.

After analysis of the expression of known CSC markers, global gene expression of the 12 isolated CSC populations was investigated. Similarly, processed datasets were used for PCA, revealing three dominant clusters among the 12 populations (Figure 4). Here, cluster one comprises all three ECSC populations and PCSCs_b. However, variances between the populations could be seen within this cluster, too. Second and biggest cluster consisted of all LCSC populations, the two remaining PCSC populations and two GSC populations. Within this cluster, LCSCs_a and LCSCs_b revealed less variances in comparison to LCSCs_c, which was clustered next to GSCs_a and GSCs_c. GSCs_b clustered independently, with the highest variance of PC1 between the group of LCSCs_a and LCSCs_b, in comparison with GSCs_b. PC2 differed with a variance of 23.73% with the greatest variation between ECSCs_c and GSCs_b (Figure 4).

**Figure 5:**
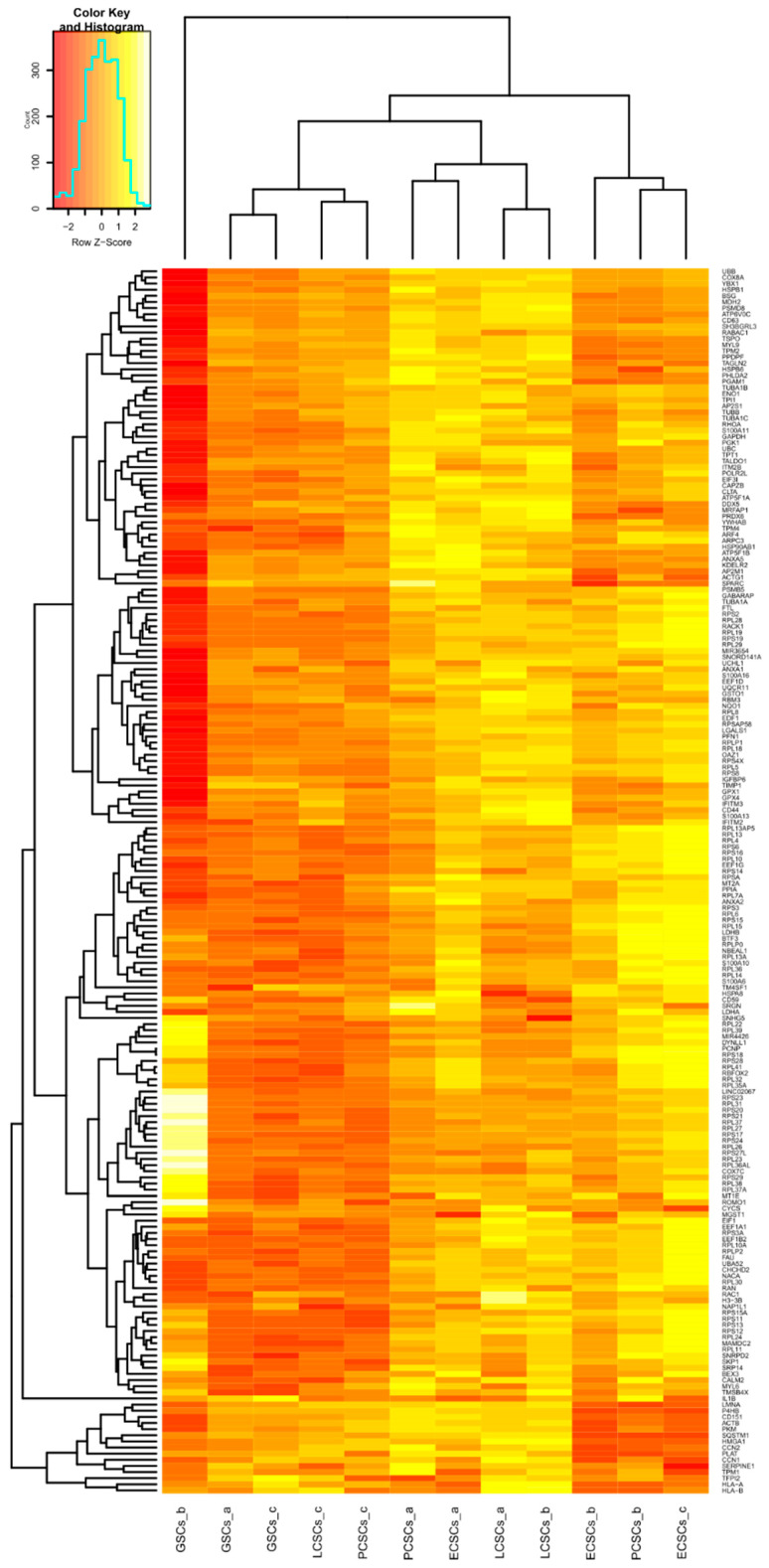
Hierarchically clustered heatmap of the 200 top expressed logarithmic gene counts within all 12 cancer stem-like cell (CSC) populations. RNA samples of different CSCs are arranged according to their corresponding expression profiles.

The large variance between the different CSC populations as well as patient-specific variations within the four distinct groups of endometrioid carcinomas, glioblastomas, and adenocarcinomas of lung and prostate, were also visible in a hierarchical clustered heatmap of the 200 top expressed genes (for all detected genes see Figure S6). Distribution of up- and down-regulated genes of the top 200 expressed genes followed the Gaussian distribution (Figure 5, for the 200 top expressed genes, which were not significantly regulated see Figure S7). Notably, tumor type-specific clustering was not observable. However, cross-group clustering emerged with GSCs_a, GSCs_c, LCSCs_c, and PCSCs_c forming one pattern. A second pattern could be seen for PCSCs_a, ECSCs_a, LCSCs_a and LCSCs_b. Further, ECSCs_b, PCSCs_b and ECSCs_c seemed to build one cluster-group (Figure 5). GSCs_b clustered independently, as already shown within the PCA (Figure 4 and Figure 5). Of note, major histocompatibility complex class I A (*HLA-A*), major histocompatibility complex class I B (*HLA-B*) and interleukin 1 beta (*IL1B*) were detected in the top 200 expressed genes among the CSC populations.

**Figure 6:**
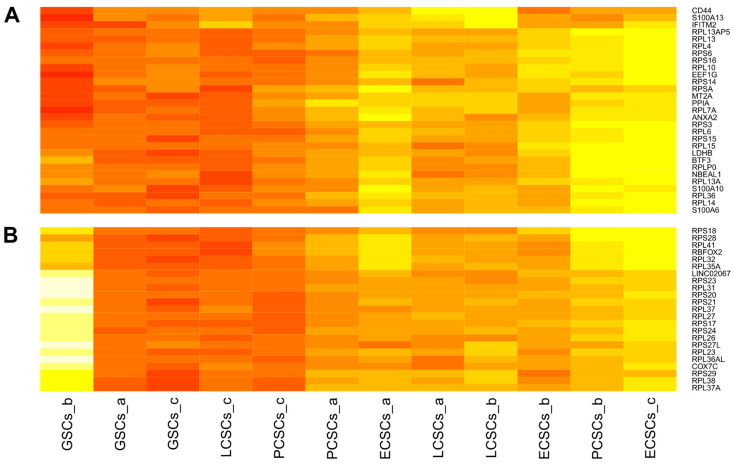
Two extracts of the hierarchically clustered heatmap of the 200 top expressed genes within all 12 cancer stem-like cell (CSC) populations. (**A**) Expressions of ribosomal biosynthesis associated genes were upregulated in endometrioid cancer stem-like cells c (ECSCs_c) and in prostate cancer stem-like cells b (PCSCs_b). Genes encoding for the S100 family were significantly expressed in all CSCs. (**B**) Expression levels of further genes relevant for ribosome biosynthesis differed between analyzed CSC populations, whereas glioblastoma stem-like cells b (GSCs_b) depicted the highest levels of genes responsible for the 40S and 60S subunit.

Next to the general clustering, it has been noticed that especially genes involved in ribosome biosynthesis have occurred more frequently (Figure 6A,B). Here, 20 different genes encoding for ribosomal proteins that are components of the 60S subunit can be found. Further, 14 genes involved in the protein synthesis of the 40S subunit of the ribosome are clustered under the 200 top expressed genes. Nevertheless, expression levels of genes relevant for the ribosome biosynthesis differed between the 12 CSC populations. Particularly striking was the high expression of diverse genes encoding for components of the 40S and 60S subunit in GSCs_b (Figure 6B). Additionally, PCSCs_b and ECSCs_c revealed high expression of ribosomal biosynthesis associated genes (Figure 6A). Among the enriched genes relevant for ribosome biosynthesis, we found distinct genes been highly expressed in GSCs_b (Figure 6A), while PCSCs_b and ECSCs_c showed other genes upregulated for ribosome biosynthesis (Figure 6B). As already shown within the dot plots, CSC marker *CD44* was ubiquitously expressed in all 12 CSC populations. Nevertheless, differences between the populations could be seen with LCSCs_a and LCSCs_b, revealing the highest expression (Figure 6A). Conspicuously, three genes of the S100 family are comprised within the top expressed genes among the 12 CSC populations, including *S100A13*, *S100A10* and *S100A6* (Figure 6A).

### 3.3. KEGG Pathway and GO-Term Analysis Reveal Broad Similarities between the Cancer Stem-like Cell Populations

**Figure 7:**
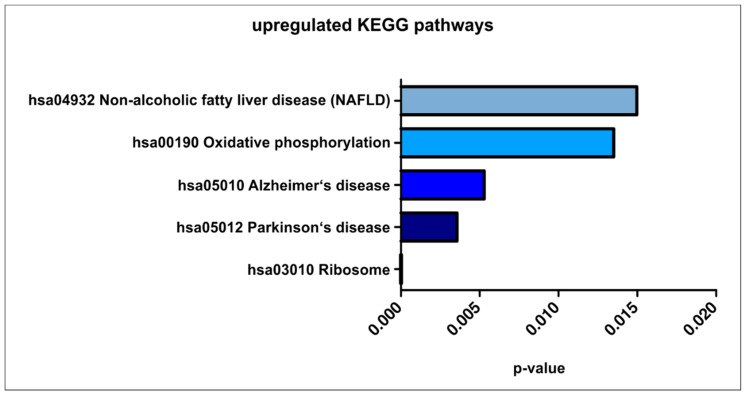
Top five upregulated KEGG pathways within the 12 investigated primary cancer stem-like cell populations (*p* < 0.05).

For an unbiased detailed analysis of the transcriptomic profiles of the 12 CSC populations, we performed a KEGG pathway analysis (Figure 7, Table S1). Here, five of the top enriched pathways were significantly overrepresented in all CSC populations and plotted together (*p* < 0.05). Next to the gene pathway responsible for “Ribosome”, which owns the strongest significance (*p* = 1.9 × 10^−41^), also “Oxidative phosphorylation” (*p* = 0.013) and “Non-alcoholic fatty liver disease” (*p* = 0.015) displayed highly enriched pathways. Surprisingly, two unexpected signaling pathways were also significantly overrepresented, namely the genes associated with “Parkinson’s disease” (*p* = 0.004) and “Alzheimer’s disease” (*p* = 0.005).

**Figure 8:**
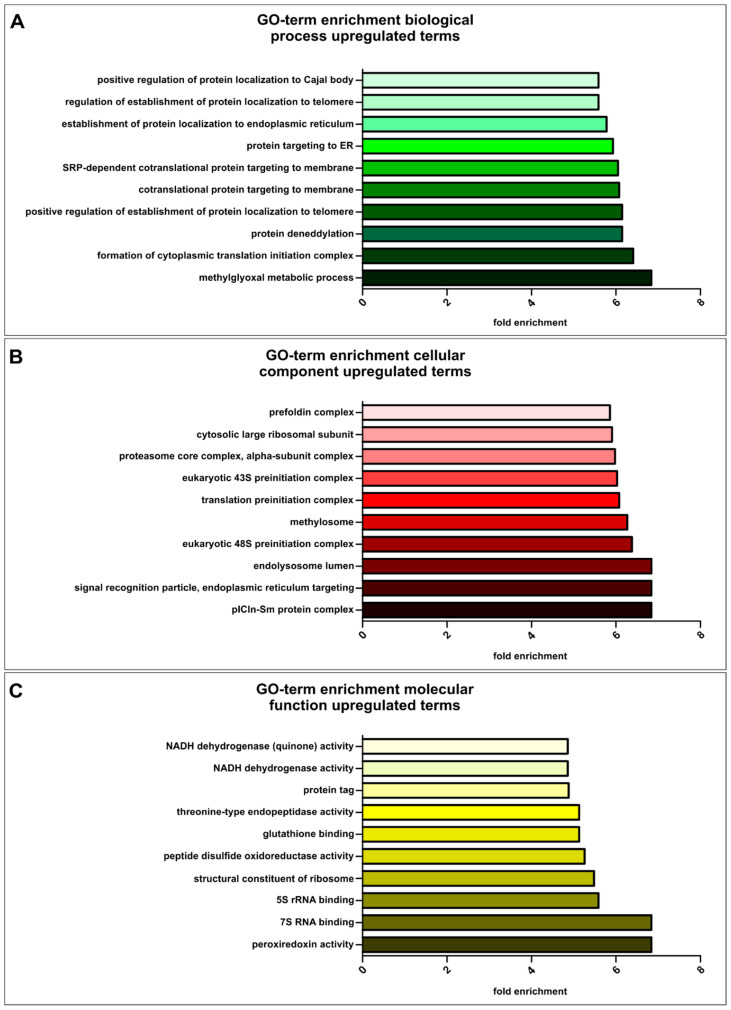
Gene ontology (GO)-term enrichment analysis of all 12 cancer stem-like cell populations, including all populations of endometrial-, glioblastoma-, lung- and prostate cancer stem-like cells, respectively. (**A**) Visualization of the top ten fold enriched GO-terms involved in biological processes (*p* < 2.00 × 10^−3^). (**B**) Top ten of the fold enriched GO-terms concerning cellular components (*p* < 1.38 × 10^−3^). **(C)** Representation of fold enriched GO-terms associated with molecular functions (*p* < 2.65 × 10^−4^).

We further conducted GO-term enrichment analysis for a more specific insight into similarly upregulated terms from biological processes, cellular components and molecular functions (Figure 8). For enriched GO-terms relating to biological processes, the top ten terms were depicted in accordance with their fold enrichments (Figure 8A, Table S2). Here, genes involved in the glycolytic pathways (fructose and glycose) were highly enriched (see “methylglyoxal metabolic process”). Further, GO-terms “formation of cytoplasmic translation initiation complex” fitting to the enriched genes in ribosomal biosynthesis and “protein deneddylation” involved in ubiquitin-mediated proteasomal degradation were upregulated. Accordingly, GO-terms relating to the cellular components (Figure 8B, Table S3) showed six-fold upregulated genes involved in “translation preinitiation complex”, “proteasome core complex, alpha-subunit complex” and “cytosolic large ribosomal subunit”. Ribosome associated GO-terms were likewise found among the terms related to molecular function, such as “7S RNA binding”, “5S rRNA binding” and “structural constituent of ribosome”. Further, genes related to “peroxiredoxin activity” were highest enriched (Figure 8C). Additionally, “NF-κB binding” was found to be upregulated in all CSC populations (Table S4).

## 4. Discussion

**Figure 9:**
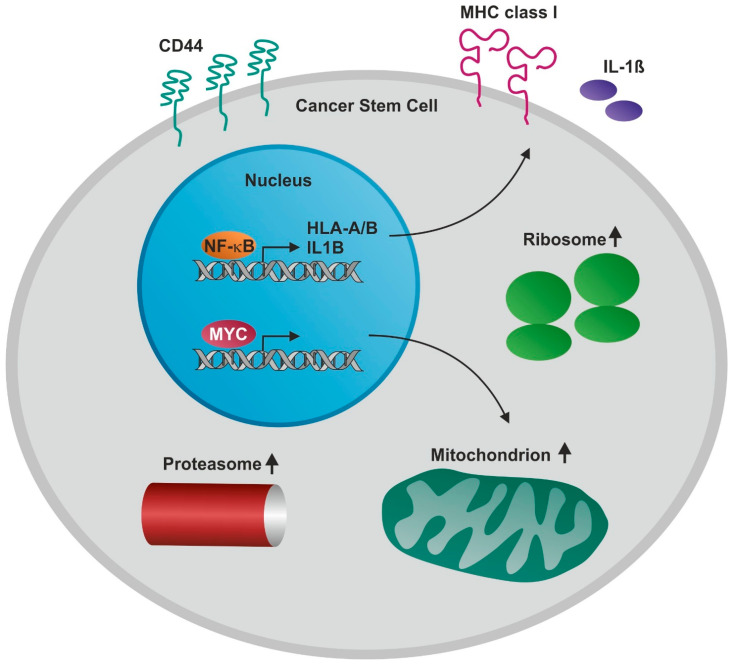
Schematic view on biological processes with enriched genes in CD44-positive cancer stem-like cells (CSCs) from glioblastoma, lung, endometrial and prostate cancer. Genes associated with the mitochondrion, proteasome, ribosome as well as *NF-κB* and *MYC* genes were highly enriched in all CSC populations.

In the present study, we report for the first time global transcriptional differences and similarities of CSCs from various tumors including glioblastoma multiforme, non-small cell lung carcinoma, endometrial carcinoma, and prostate carcinoma. We found the transcripts of all investigated CSC-populations to share significantly upregulated genes associated with the mitochondrion, proteasome, and ribosome (Figure 9).

In 1924, Otto Warburg and co-workers published a seminal paper on aerobic glycolysis in rat tumor cells and sections derived from human tumors [68]. The Warburg effect described an increased rate of glucose fermentation leading to lactic acid by tumor cells, even under aerobic conditions [69]. Eight-seven years later, the Warburg effect was included in the list of general hallmarks of cancer. Tumor cells are able to reprogram the energy metabolism to aerobic glycolysis independent to mitochondrial function to produce adenosine triphosphate (ATP) [70]. However, a tumor is increasingly recognized to consist of a highly heterogeneous cell mass, with the rare cell type of CSCs driving metastasis, invasiveness, therapeutic resistance and recurrence of the tumor (reviewed in [71]). Here, we determined the global transcriptomes of CSCs propagated in vitro. Extending the findings of Warburg, these cells seem to have high metabolic plasticity and might use mitochondria for oxidative phosphorylation. In this line, gene enrichment analysis of the genes expressed in CSCs in comparison to the human genome showed a more than five-fold enrichment of expressed genes involved in mitochondria and oxidative phosphorylation. Accordingly, the amount of metabolic flexibility allowing the switch from oxidative phosphorylation to aerobic glycolysis was reported for CSCs (reviewed in [72]). In particular, a recent study investigated whether the metabolic state (especially the Warburg effect) of GSCs differs from the bulk of the tumor cells with a newly developed imaging system [73]. Vlashi and coworkers reported that monolayer cultures of GSCs produce about 20% less ATP than neurosphere cultures. Of note, another study showed high expressions of CD133 and Nestin in cultured GSC spheres but not in non-sphere GSC monolayers, cultured in 10% FBS [74]. In our study, we detected no difference in the expression of CSC markers in sphere cultures in comparison to adherently grown CSC populations. This might be due to a different experimental strategy used here, as we selected CSCs by differential trypsinization and cultured adherent populations with 10% serum supplemented with B27, bFGF-2 and EGF. Using specific inhibitors for oxidative phosphorylation and aerobic glycolysis, the authors suggested that ATP in both monolayer and neurosphere cultures was mainly produced by glycolysis and oxidative phosphorylation. Inhibition of glycolysis could be compensated through increased oxidative phosphorylation and vice versa. However, the authors conclude that the GSC-metabolism mainly relies on oxidative phosphorylation, which is in line to the here observed enrichment in genes involved in mitochondria and oxidative phosphorylation. Of note, mutations in *IDH1* and *IDH2* genes were shown to reprogram the metabolism of cancer cells to produce the onco-metabolite D-2-hydroxyglutarate and cells with mutations might be additionally dependent on lactate (see for review [75]). In our analysis, CSCs from a secondary GBM showing an *IDH1* mutation (see Figure 4, GSCs_b) did not cluster with the other analyzed GBM-derived CSCs without *IDH1* mutation because of significant changes in global gene expression.

Otto Warburg had many important contributions to biochemistry, such as the discovery of the ‘oxygen-transferring ferment of respiration’ (cytochrome-c oxidase complex), leading to him receiving the Nobel Prize in physiology or medicine, 1931. We found expressed genes enriched, which might be important for oxidative phosphorylation in mitochondria, such as NADH dehydrogenase activity, proton-transporting ATP synthase activity and cytochrome-c oxidase activity. Since respiratory chain activity leads to constant production of the reactive oxygen intermediate (ROS) it might not be surprising to find enrichment of genes, which code for antioxidant activity such as peroxiredoxin activity, glutathione binding and glutathione peroxidase activity. Some of the ROS produced might result in NF-κB binding. Other organelles containing enriched genes include ribosomes and 26 proteasomes. Indeed, oxidative phosphorylation in mitochondria have been identified as hallmark of CSC metabolism (reviewed in [76]). We also analyzed the expression of general CSC markers (Figure S7). Of special interest might be our recent observation of MYC, as a potential regulator of survival in colon carcinoma-specific CSCs (see [42]). In our study, we could detect MYC expressions in each investigated CSC population. Furthermore, published cancer surface markers CD44 and CD59, as well as the insulin-like growth factor binding protein 2 (IGFBP2), were expressed in all analyzed CSCs. In this context, Chen and Ding demonstrated gradually increased CD59 expression levels in different breast- and lung parental cancer cell lines and further observed that the absence of CD59 resulted in a completely suppression of tumorigenesis in vivo [77]. These results were investigated within sphere cultures, whereas we also detected CD59 within our adherently cultured CSC populations, further substantiating stem-like characteristics in our CSC models. Next, gene expressions for ribosomal proteins like *RPLP1*, *RPS27L*, *RPL14* and *RPL21* as well as the nuclear encoded and mitochondrial localized protein *COX8A* were detected after transcriptomic analysis. Finally, genes involved in cell cycle regulation and tumor growth like *CDK16*, *CDC20* and the Ras homologue enriched in brain (*RHEB)* were ubiquitously expressed in our established CSCs. In this context, RHEB is commonly known to function in human carcinogenesis [78]. In more detail, Tian and co-workers reported the inhibition of cell proliferation and initiation of apoptosis in colorectal cancer cells via silencing of the *RHEB* gene [79].

We have previously shown that the guanine exchange factor (GEF) pleckstrin homology and RhoGEF domain containing G5 (*PLEKHG5*) plays an essential role in the formation of autolysosomes in glioblastoma cells and induces the transcription factor NF-κB [80]. However, in the present dataset we could not detect any expression of *PLEKHG5*. With respect to a recently proposed role of NF-κB in cancer and CSCs (reviewed in [1,81]), we searched for evidence of activated NF-κB in this transcriptome dataset. We detected highly enriched genes involved in the GO-term “NF-κB binding”. However, out of all five DNA-binding subunits, only the expression of *RELA* in all CSC samples was detectable. Furthermore, expression of interleukin 6 and tumor necrosis factor-alpha was not detectable in CSCs, whereas we observed NF-κB target gene expression of *IL1B*, *HLA-A* as well as *HLA-B* in all CSC population. We therefore conclude that further experiments might be necessary to provide conclusive data on the role of NF-κB in CSCs.

We further demonstrate high amounts of ribosome-associated gene expressions in CSCs independent of the tumor type. These data suggest a mechanism of ribosome-enrichment for oxidative phosphorylation in CSCs. On the mechanistic level, RNA polymerase III (Pol III) may transcribe the ribosomal 5S rRNA and the 5.8S, 18S as well as 28S rRNAs are known to arise from the processing of a common precursor rRNA (47S) transcribed by the Pol I. Accordingly, altered ribosome components were discussed to play a significant role in CSCs [82]. There is additional evidence that ribosome biosynthesis and protein synthesis might be directed by transcription factor *MYC* (reviewed in [83]). Proteasome-related transcription was enriched by more than a factor of six in CSCs, although investigations with fluorogenic proteasome substrates suggest a low activity of proteasomal protein degradation in CSCs of breast cancer and glioma [84].

Limitations: on a technical level, this study relies on in vitro protein and transcriptomic data generated with newly developed nanopore sequencing. Although we generally achieved full-length transcript data using this technology, read depth is not as high as in standard Illumina approaches, but seems to be sufficiently sensitive to detect differences and similarities between CSC populations. However, due to the performed read depth, we cannot exclude the possibility of missing low abundant transcripts as transcription factors.

## 5. Conclusions

From our transcriptomic analysis, we conclude that the here used CSC populations could be divided into three clusters independent of the parental tumor. Nevertheless, we detected conserved expressions of the CSC markers CD44, Nestin, and MYC on mRNA and protein level as well as transcriptional expression of *CD59*. Moreover, GO-term and KEGG pathway enrichment analysis revealed upregulated genes related to ribosomal biosynthesis, the mitochondrion, oxidative phosphorylation, and glycolytic pathways as well as to the proteasome, suggesting the great extent of metabolic flexibility in CSCs. Taken together, transcriptomic analysis might pave the way for future pathway-directed therapies such as targeting mitochondria and 80S ribosomes, together with the already established proteasome. However, further in vitro and in vivo studies are necessary to overcome potential limitations of the study and for specific target discovery.

## Figures and Tables

**Table 1:** Cell population-specific donor information.

Donor of Cell Population	Tumor Typing/Characterization	WHO Grade	Sex	Age
ECSCs_a	Endometrioid carcinoma of the corpus uteri	GII	female	72
ECSCs_b	Endometrioid carcinoma of the corpus uteri	GI	female	83
ECSCs_c	Endometrioid carcinoma of the corpus uteri with invasion of the outer half of the myometrium and invasion of the cervix uteri	GII	female	86
GSCs_a	Primary glioblastoma multiforme, *IDH1* wildtype with *MGMT* promoter methylation	GIV	female	60
GSCs_b	Secondary glioblastoma multiforme, *IDH1* mutation with *MGMT* promoter methylation	GIV	male	42
GSCs_c	Primary glioblastoma multiforme, *IDH1* wildtype without *MGMT* promoter methylation	GIV	male	69
LCSCs_a	Highly metastatic adenocarcinoma of the lung, *EGFR* mutation	n.a.	female	50
LCSCs_b	Multifocal adenocarcinoma of the lung	GII	female	61
LCSCs_c	Adenocarcinoma of the lung, *KRAS* and *STK11* mutation	GII	female	49
PCSCs_a	Acinar adenocarcinoma	GIII	male	71
PCSCs_b	Acinar adenocarcinoma	GII	male	57
PCSCs_c	Locally advanced acinar adenocarcinoma	GV	male	72

## Data Availability

The transcriptomic data are available via NCBI BioProject PRJNA697831.

## Supplementary Materials

The following are available online at https://www.mdpi.com/2072-6694/13/5/1136/s1, Figure S1: Immunocytochemical stainings of cultured cancer stem-like cells from one population of endometrioid cancer and glioblastoma multiforme, grown as spheres, Figure S2: Co-expressions of CD44/CD133 in cancer stem-like cell populations after immunocytochemistry, Figure S3: Nestin protein expressions of adherently grown cancer stem-like cell populations, detected via immunocytochemical stainings, Figure S4: MYC immunocytochemistry of adherently cultured cancer stem-like cell populations, Figure S5: Immunostaining of CD44/CD133, Nestin and MYC in cultured adult human dermal fibroblasts (HDFs) as biological negative control to primary isolated cancer stem-like cell populations., Figure S6: Heatmap of all normalized gene counts detected by nanopore sequencing, Figure S7: Heatmap of the 200 top expressed, not significantly regulated genes detected via nanopore sequencing, Table S1: KEGG pathway analysis, Table S2: GO-term enrichment in biological processes, Table S3: GO-term enrichment in cellular components, Table S4: GO-term enrichment of molecular functions.
