# Supplementary material for: Nanopore Sequencing Reveals Global Transcriptome Signatures of Mitochondrial and Ribosomal Gene Expressions in Various Human Cancer Stem-like Cell Populations
Source: Cancers (Basel). 2021 Mar 6;13(5):1136. doi: 10.3390/cancers13051136 (PMC7962028; doi:10.3390/cancers13051136)
Supplement: Supplementary file 1 [file cancers-13-01136-s001.pdf]

cancers

MDPI

Supplementary Materials

Nanopore Sequencing Reveals Global Transcriptome Signatures of Mitochondrial and Ribosomal Gene Expressions in Various Human Cancer Stem-like Cell Populations

Kaya E. Witte, Oliver Hertel, Beatrice A. Windmöller, Laureen P. Helweg, Anna L. Höving, Cornelius Knabbe, Tobias Busche, Johannes F. W. Greiner, Jörn Kalinowski, Thomas Noll, Fritz Mertzlufft, Morris Beshay, Jesco Pfitzenmaier, Barbara Kaltschmidt, Christian Kaltschmidt, Constanze Banz-Jansen and Matthias Simon

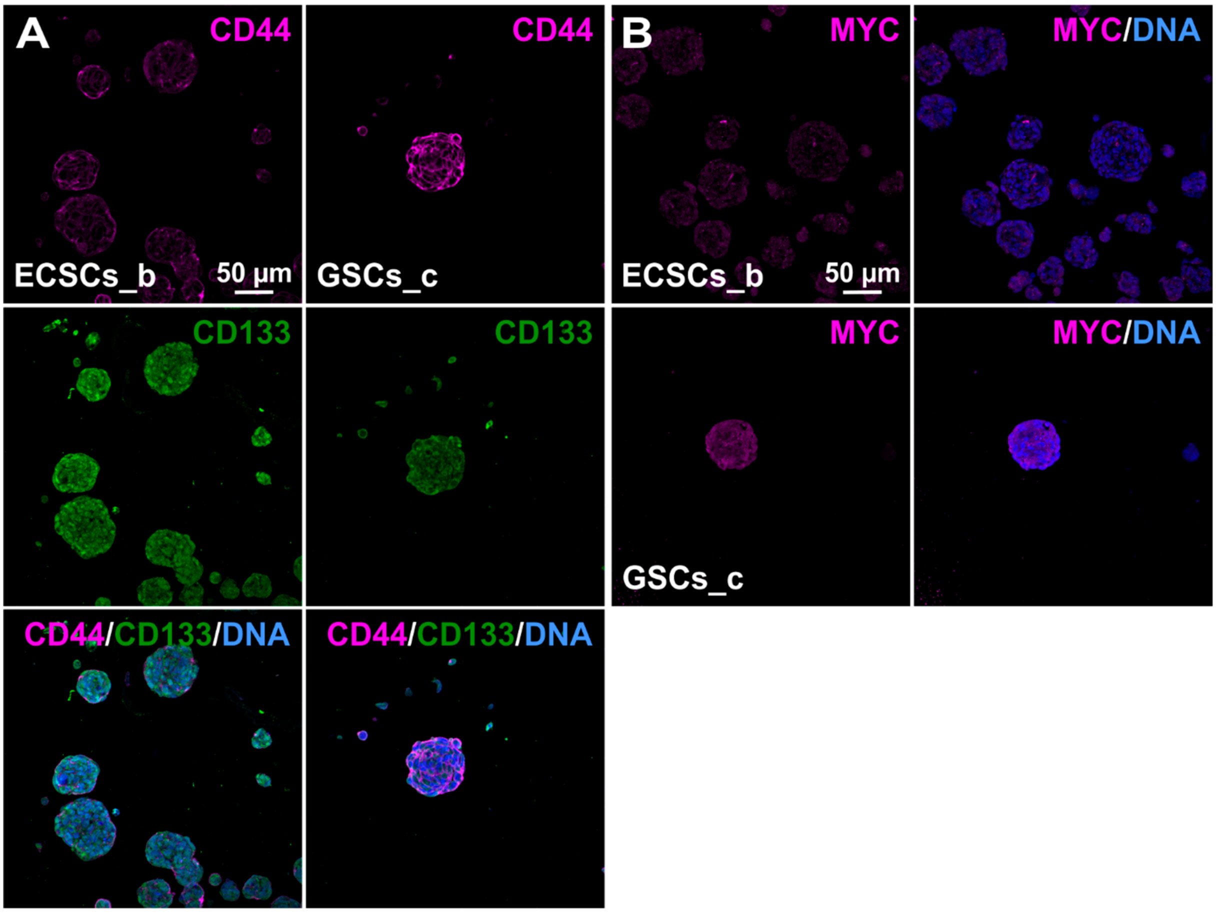

Figure S1. Immunocytochemical stainings of cultured cancer stem-like cells from one population of endometrioid cancer and glioblastoma multiforme, grown as spheres.

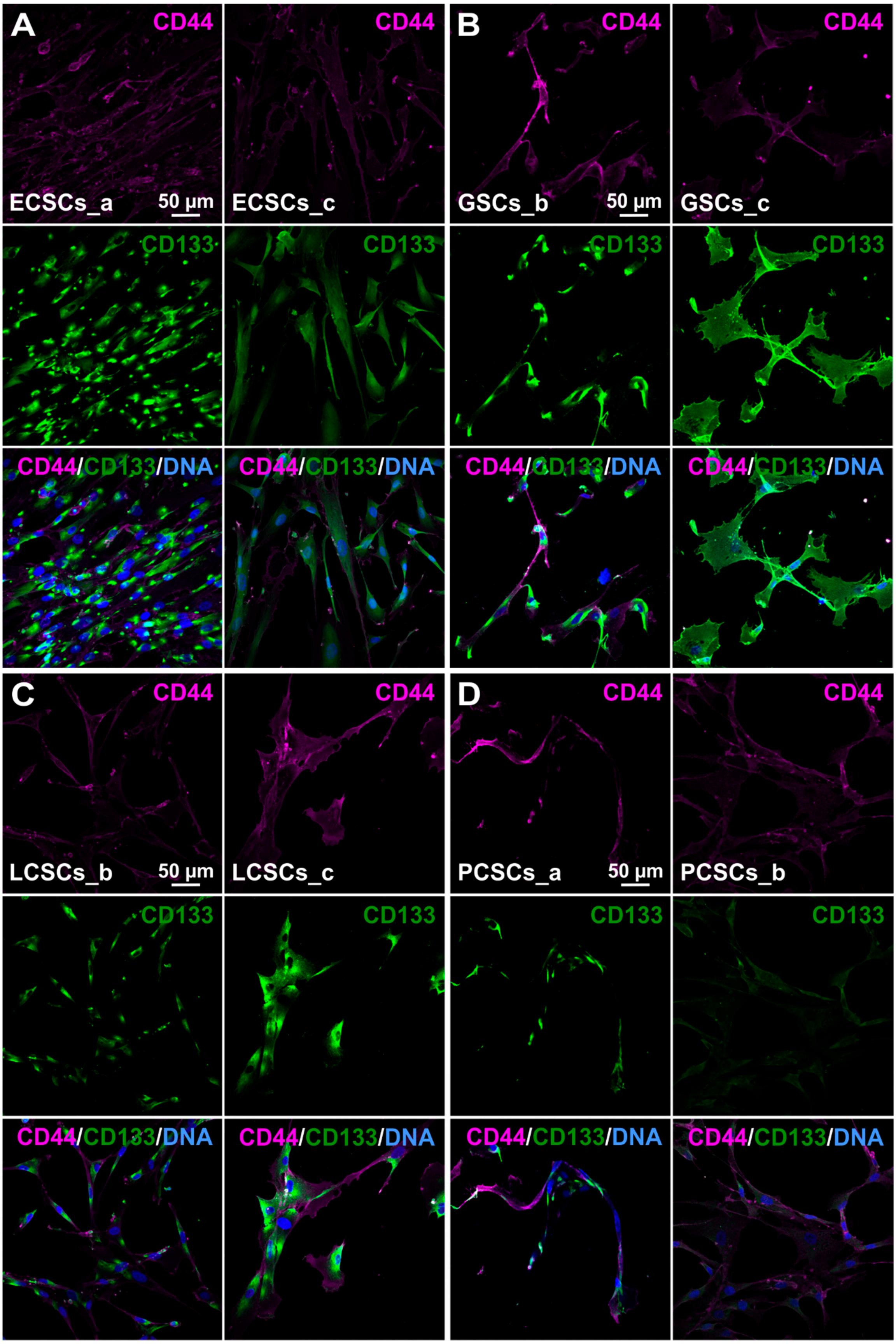

Figure S2. Co-expressions of CD44/CD133 in cancer stem-like cell populations after immunocytochemistry.

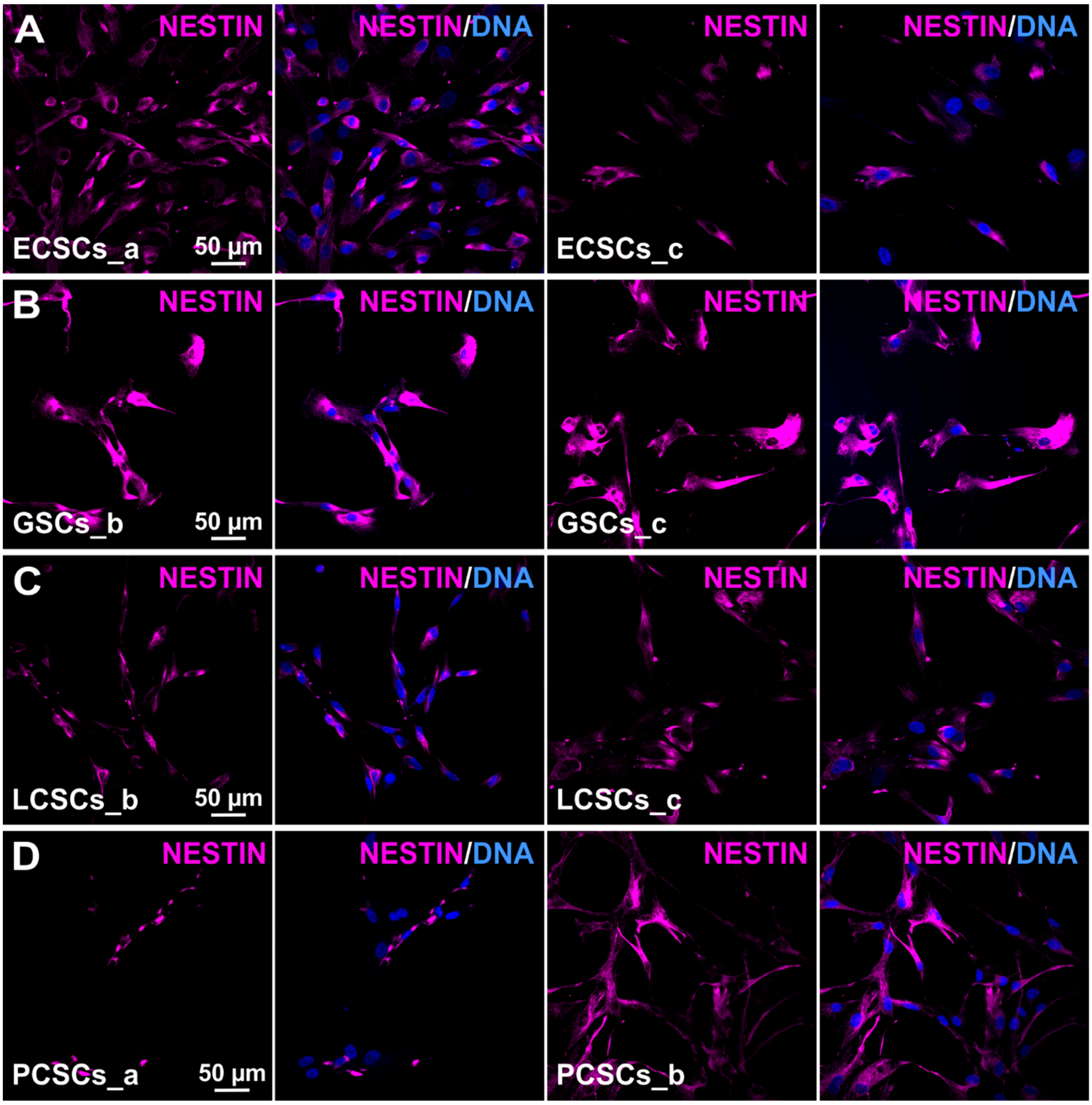

Figure S3. Nestin protein expressions of adherently grown cancer stem-like cell populations, detected via immunocytochemical stainings.

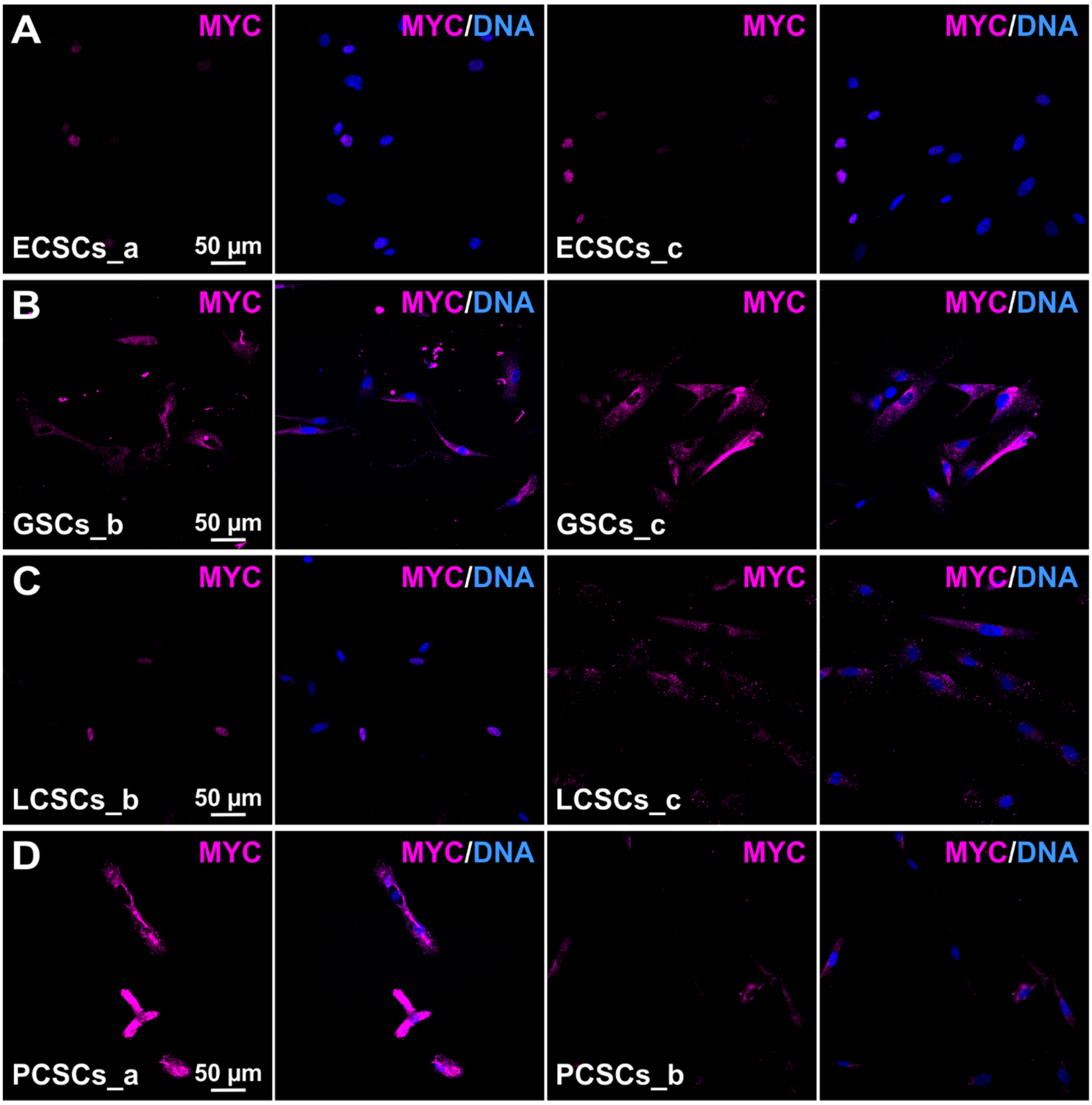

Figure S4. MYC immunocytochemistry of adherently cultured cancer stem-like cell populations.

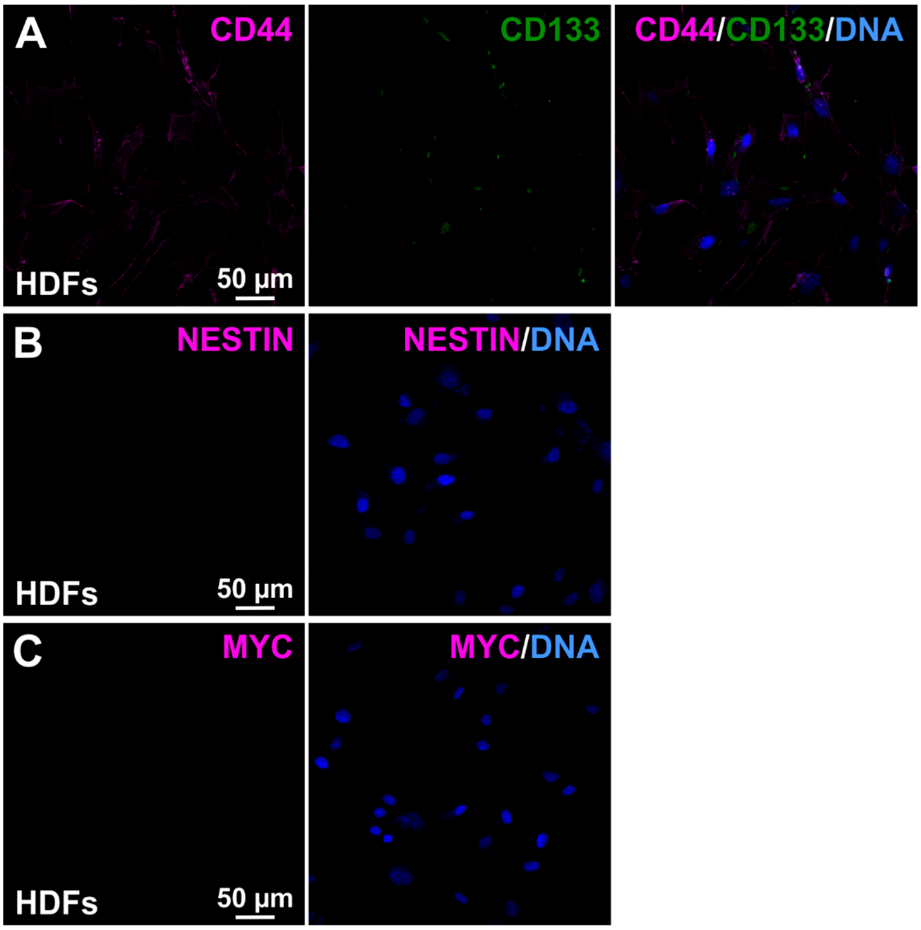

Figure S5. Immunostaining of CD44/CD133, Nestin and MYC in cultured adult human dermal fibroblasts (HDFs) as biological negative control to primary isolated cancer stem-like cell populations.

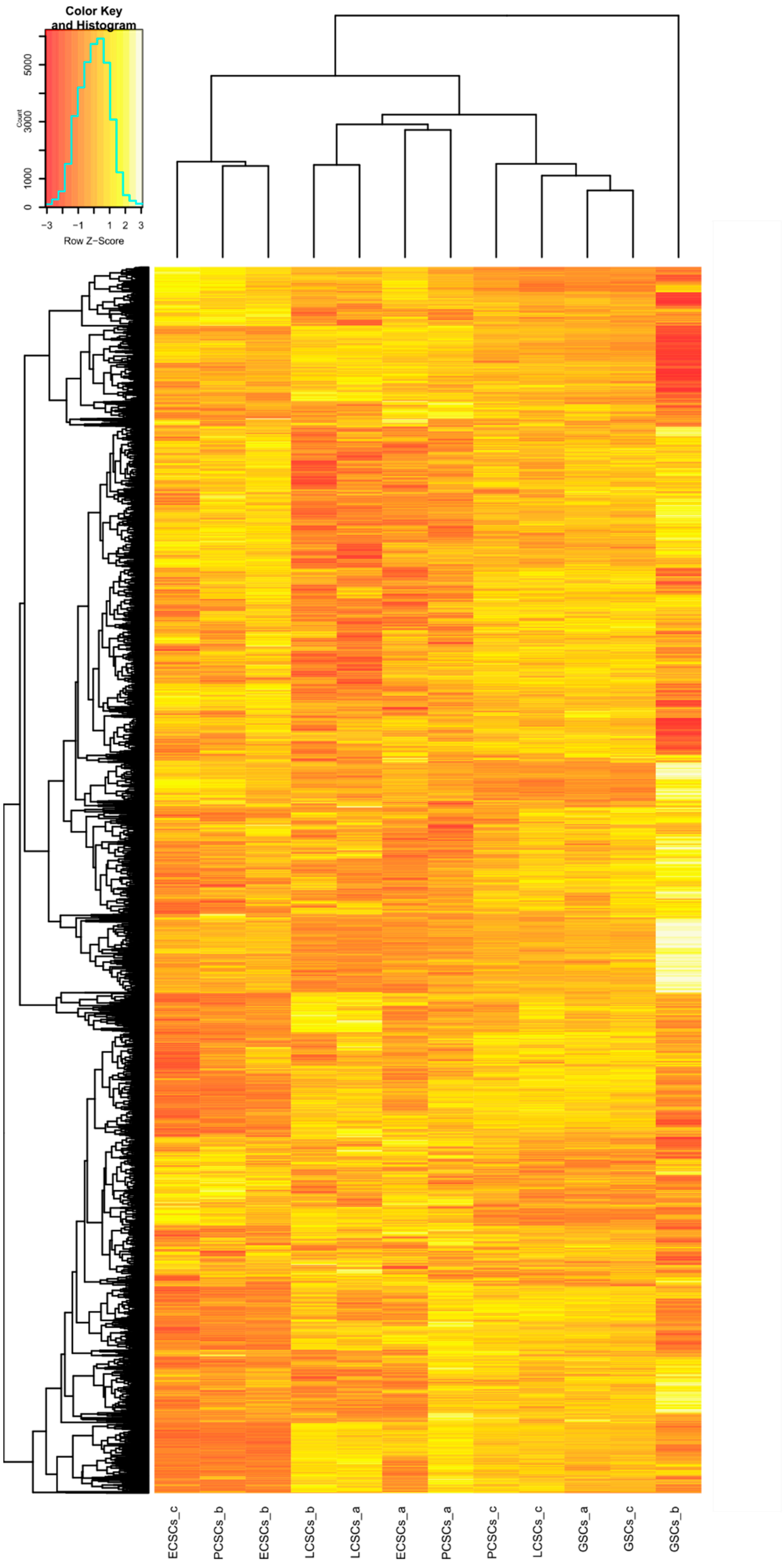

Figure S6. Heatmap of all normalized gene counts detected by nanopore sequencing.

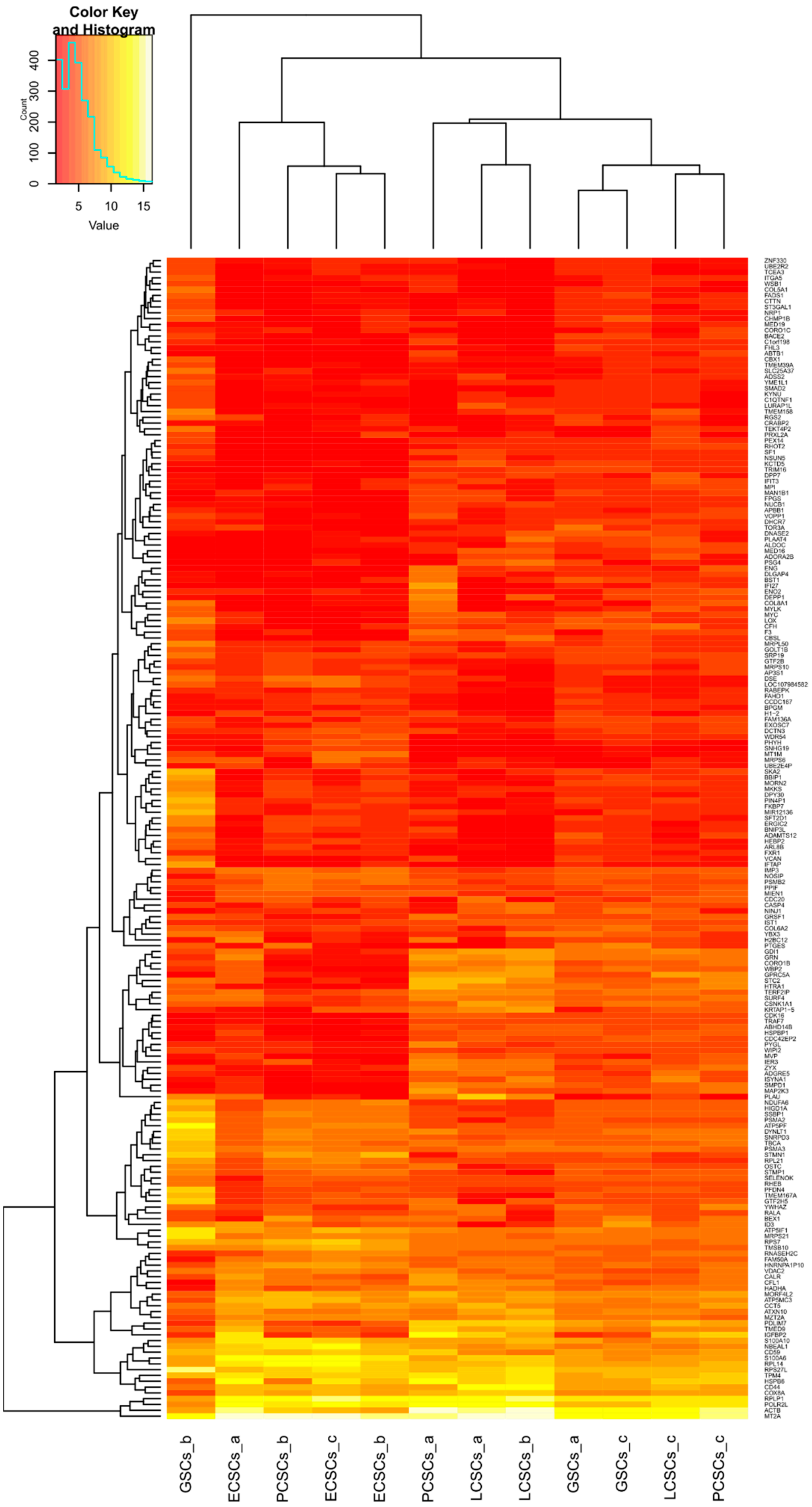

Figure S7. Heatmap of the 200 top expressed, not significantly regulated genes detected via nanopore sequencing.

Table S1. KEGG pathway analysis.

KEGG pathway	*p*-Value	Counts
hsa03010:Ribosome	1.9165×10^{-41}	114
hsa05012:Parkinson's disease	0.00356609	77
hsa00190:Oxidative phosphorylation	0.01349267	73
hsa05010:Alzheimer's disease	0.00528544	84
hsa05016:Huntington's disease	0.1514617	91
hsa04932:Non-alcoholic fatty liver disease (NAFLD)	0.01495838	76
hsa03050:Proteasome	0.38959908	35
hsa01130:Biosynthesis of antibiotics	125.001549	92
hsa01100:Metabolic pathways	3848.90259	340
hsa03040:Spliceosome	388599.824	61
hsa04141:Protein processing in endoplasmic reticulum	1138676.51	71
hsa01200:Carbon metabolism	3811133.63	51
hsa00480:Glutathione metabolism	7058345.97	30
hsa03060:Protein export	3742945.53	17
hsa04142:Lysosome	$1.2718 \times 10^{+10}$	48
hsa01230:Biosynthesis of amino acids	219465948	33
hsa00520:Amino sugar and nucleotide sugar metabolism	$3.266 \times 10^{+10}$	25
hsa05131:Shigellosis	$4.2601 \times 10^{+10}$	28
hsa03008:Ribosome biogenesis in eukaryotes	$8.3231 \times 10^{+10}$	34
hsa00620:Pyruvate metabolism	$8.5255 \times 10^{+10}$	20
hsa00010:Glycolysis / Gluconeogenesis	$1.1037 \times 10^{+12}$	28
hsa00020:Citrate cycle (TCA cycle)	$2.3337 \times 10^{+12}$	16
hsa03013:RNA transport	$2.5177 \times 10^{+11}$	55
hsa00270:Cysteine and methionine metabolism	$4.8801 \times 10^{+11}$	18
hsa00240:Pyrimidine metabolism	$8.8373 \times 10^{+11}$	35
hsa05130:Pathogenic Escherichia coli infection	$1.2563 \times 10^{+16}$	21
hsa00062:Fatty acid elongation	$1.5616 \times 10^{+16}$	13
hsa05110:Vibrio cholerae infection	$1.6629 \times 10^{+15}$	21
hsa03020:RNA polymerase	$1.9781 \times 10^{+16}$	15
hsa04260:Cardiac muscle contraction	$2.1494 \times 10^{+16}$	27
hsa05132:Salmonella infection	$2.3488 \times 10^{+16}$	29
hsa00330:Arginine and proline metabolism	$6.3395 \times 10^{+15}$	19
hsa04145:Phagosome	$6.8854 \times 10^{+14}$	44
hsa00280:Valine, leucine and isoleucine degradation	$7.4667 \times 10^{+15}$	18
hsa03420:Nucleotide excision repair	$7.4667 \times 10^{+15}$	18
hsa00052:Galactose metabolism	$9.6388 \times 10^{+15}$	13
hsa05134:Legionellosis	$1.516 \times 10^{+16}$	19
hsa05203:Viral carcinogenesis	$1.6536 \times 10^{+16}$	55
hsa04110:Cell cycle	$1.76 \times 10^{+15}$	36
hsa04144:Endocytosis	$1.7885 \times 10^{+16}$	63
hsa03030:DNA replication	$1.8504 \times 10^{+16}$	14
hsa00030:Pentose phosphate pathway	$2.0013 \times 10^{+15}$	12
hsa00230:Purine metabolism	$2.9032 \times 10^{+16}$	47
hsa05133:Pertussis	$3.5081 \times 10^{+15}$	23
hsa00260:Glycine, serine and threonine metabolism	$3.607 \times 10^{+16}$	14
hsa05169:Epstein-Barr virus infection	$3.7298 \times 10^{+15}$	34
hsa00640:Propanoate metabolism	$3.9673 \times 10^{+16}$	11
hsa00051:Fructose and mannose metabolism	$4.1736 \times 10^{+15}$	12
hsa05100:Bacterial invasion of epithelial cells	$5.2538 \times 10^{+16}$	23
hsa00510:N-Glycan biosynthesis	$5.261 \times 10^{+15}$	16
hsa05323:Rheumatoid arthritis	$6.2837 \times 10^{+14}$	25
hsa04115:p53 signaling pathway	$6.5035 \times 10^{+14}$	20
hsa00410:beta-Alanine metabolism	$7.5734 \times 10^{+15}$	11
hsa04962:Vasopressin-regulated water reabsorption	$8.7169 \times 10^{+15}$	14
hsa04978:Mineral absorption	$8.7169 \times 10^{+15}$	14

Table S2. GO-term enrichment in biological processes.

Go-Term	Fold Enrichment	Raw *p*–Value
methylglyoxal metabolic process	6.84	2.00×10^{-03}
formation of cytoplasmic translation initiation complex	6.41	1.50×10^{-06}

protein deneddylation	6.15	2.45×10^{-04}
positive regulation of establishment of protein localization to telomere	6.15	2.45×10^{-04}
cotranslational protein targeting to membrane	6.08	5.57×10^{-31}
SRP-dependent cotranslational protein targeting to membrane	6.05	1.54×10^{-29}
protein targeting to ER	5.93	2.33×10^{-33}
establishment of protein localization to endoplasmic reticulum	5.78	4.36×10^{-33}
regulation of establishment of protein localization to telomere	5.59	3.95×10^{-04}
positive regulation of protein localization to Cajal body	5.59	3.95×10^{-04}
regulation of protein localization to Cajal body	5.59	3.95×10^{-04}
regulation of ER-associated ubiquitin-dependent protein catabolic process	5.47	9.28×10^{-04}
positive regulation of telomerase RNA localization to Cajal body	5.47	4.95×10^{-05}
negative regulation of ubiquitin protein ligase activity	5.47	9.28×10^{-04}
mitochondrial electron transport, ubiquinol to cytochrome c	5.37	1.14×10^{-04}
translational initiation	5.32	6.08×10^{-35}
GDP-mannose metabolic process	5.32	2.19×10^{-03}
proteasomal ubiquitin-independent protein catabolic process	5.28	1.89×10^{-06}
protein localization to endoplasmic reticulum	5.27	2.15×10^{-34}
positive regulation of protein localization to chromosome, telomeric region	5.26	2.65×10^{-04}
mitochondrial ATP synthesis coupled proton transport	5.21	4.31×10^{-06}
cytoplasmic translation	5.19	3.94×10^{-18}
regulation of establishment of protein localization to chromosome	5.13	6.14×10^{-04}
nucleotide-excision repair, DNA damage recognition	5.05	2.90×10^{-06}
mitochondrial translational elongation	5.05	5.12×10^{-20}
regulation of protein localization to chromosome, telomeric region	5.01	1.76×10^{-04}
viral translation	4.94	5.08×10^{-05}
viral transcription	4.89	1.61×10^{-24}
nuclear-transcribed mRNA catabolic process, nonsense-mediated decay	4.84	7.24×10^{-25}
mitochondrial translational termination	4.84	8.73×10^{-19}
mitochondrial electron transport, NADH to ubiquinone	4.74	1.24×10^{-10}
cellular response to nitrogen starvation	4.73	9.22×10^{-04}
PERK-mediated unfolded protein response	4.73	9.22×10^{-04}
cellular response to nitrogen levels	4.73	9.22×10^{-04}
regulation of cellular amino acid metabolic process	4.69	9.24×10^{-14}
NADH dehydrogenase complex assembly	4.66	2.04×10^{-13}
mitochondrial respiratory chain complex I assembly	4.66	2.04×10^{-13}
cytoplasmic translational initiation	4.63	5.65×10^{-07}
mitochondrial ATP synthesis coupled electron transport	4.61	3.07×10^{-17}
translational termination	4.58	1.85×10^{-18}
regulation of telomerase RNA localization to Cajal body	4.56	1.70×10^{-04}
ATP synthesis coupled electron transport	4.56	4.49×10^{-17}
ribosomal small subunit assembly	4.56	4.93×10^{-05}
ATP synthesis coupled proton transport	4.47	9.42×10^{-06}
energy coupled proton transport, down electrochemical gradient	4.47	9.42×10^{-06}
protein targeting to membrane	4.44	8.66×10^{-32}
viral budding via host ESCRT complex	4.44	1.10×10^{-04}
translational elongation	4.43	5.50×10^{-22}
midbody abscission	4.42	3.82×10^{-04}
multivesicular body organization	4.41	1.82×10^{-06}
antigen processing and presentation of exogenous peptide antigen via MHC class I, TAP-dependent	4.41	8.29×10^{-14}
polyamine biosynthetic process	4.39	1.34×10^{-03}
proteasome assembly	4.39	1.34×10^{-03}
mitochondrial translation	4.37	3.21×10^{-19}
glutathione derivative biosynthetic process	4.35	7.13×10^{-05}
glutathione derivative metabolic process	4.35	7.13×10^{-05}
nucleotide-excision repair, DNA duplex unwinding	4.35	7.13×10^{-05}
oxidative phosphorylation	4.33	2.74×10^{-20}
multivesicular body assembly	4.33	4.00×10^{-06}
viral gene expression	4.32	3.45×10^{-28}
regulation of transcription from RNA polymerase II promoter in response to hypoxia	4.29	1.70×10^{-13}
anaphase-promoting complex-dependent catabolic process	4.29	1.02×10^{-14}
integrated stress response signaling	4.27	8.56×10^{-04}
protein insertion into ER membrane	4.27	4.60×10^{-05}
nucleotide-excision repair, preincision complex assembly	4.24	8.77×10^{-06}
mitotic cytokinetic process	4.23	1.58×10^{-04}

nucleotide-excision repair, DNA incision, 3'-to lesion	4.23	1.58×10^{-04}
nucleotide-excision repair, preincision complex stabilization	4.23	1.58×10^{-04}
chaperone-mediated protein complex assembly	4.23	1.58×10^{-04}
antigen processing and presentation of exogenous peptide antigen via MHC class I	4.22	1.58×10^{-13}
respiratory electron transport chain	4.16	2.04×10^{-17}
mitochondrial electron transport, cytochrome c to oxygen	4.10	3.48×10^{-04}
S phase	4.10	1.34×10^{-10}
mitotic S phase	4.10	1.34×10^{-10}
protein import into mitochondrial matrix	4.10	3.48×10^{-04}
aerobic electron transport chain	4.10	3.48×10^{-04}
protein maturation by iron-sulfur cluster transfer	4.10	1.91×10^{-03}
translation	4.09	2.66×10^{-56}
mitochondrial respiratory chain complex assembly	4.05	1.44×10^{-15}
negative regulation of ubiquitin-protein transferase activity	4.02	1.21×10^{-03}

Table S3. GO-term enrichment in cellular components.

Go-Term	Fold Enrichment	Raw *p*–Value
pICln-Sm protein complex	6.84	2.00×10^{-03}
signal recognition particle, endoplasmic reticulum targeting	6.84	2.00×10^{-03}
endolysosome lumen	6.84	4.87×10^{-03}
eukaryotic 48S preinitiation complex	6.38	3.49×10^{-06}
methylosome	6.27	4.43×10^{-05}
translation preinitiation complex	6.08	1.08×10^{-06}
eukaryotic 43S preinitiation complex	6.03	2.49×10^{-06}
proteasome core complex, alpha-subunit complex	5.98	1.38×10^{-03}
cytosolic large ribosomal subunit	5.91	5.49×10^{-18}
prefoldin complex	5.86	3.31×10^{-03}
U7 snRNP	5.86	3.31×10^{-03}
eukaryotic translation initiation factor 3 complex, eIF3m	5.86	3.31×10^{-03}
proteasome core complex	5.81	7.59×10^{-07}
mitochondrial proton-transporting ATP synthase complex, coupling factor F(o)	5.70	1.69×10^{-04}
cytosolic small ribosomal subunit	5.70	4.65×10^{-14}
eukaryotic translation initiation factor 3 complex	5.63	9.28×10^{-06}
Proteasome core complex, beta-subunit complex	5.59	3.95×10^{-04}
cytosolic ribosome	5.49	4.73×10^{-29}
proteasome accessory complex	5.47	1.60×10^{-07}
ribosomal subunit	5.33	2.95×10^{-46}
signal recognition particle	5.32	2.19×10^{-03}
small ribosomal subunit	5.31	5.03×10^{-19}
proteasome regulatory particle	5.28	1.89×10^{-06}
large ribosomal subunit	5.27	1.95×10^{-28}
RNA polymerase I complex	5.26	2.65×10^{-04}
proteasome regulatory particle, lid subcomplex	5.13	5.17×10^{-03}
proteasome regulatory particle, base subcomplex	5.13	6.14×10^{-04}
U4 snRNP	4.97	1.42×10^{-03}
chaperonin-containing T-complex	4.97	1.42×10^{-03}
chaperone complex	4.92	1.94×10^{-06}
respiratory chain complex I	4.92	1.67×10^{-11}
NADH dehydrogenase complex	4.92	1.67×10^{-11}
mitochondrial respiratory chain complex I	4.92	1.67×10^{-11}
proton-transporting ATP synthase complex, coupling factor F(o)	4.88	4.02×10^{-04}
TIM23 mitochondrial import inner membrane translocase complex	4.88	4.02×10^{-04}
polysomal ribosome	4.82	5.10×10^{-08}
autolysosome	4.78	3.31×10^{-03}
organellar ribosome	4.76	2.89×10^{-18}
mitochondrial ribosome	4.76	2.89×10^{-18}
ribosome	4.76	5.10×10^{-47}
organellar small ribosomal subunit	4.64	1.91×10^{-06}
mitochondrial small ribosomal subunit	4.64	1.91×10^{-06}
organellar large ribosomal subunit	4.60	1.10×10^{-11}
mitochondrial large ribosomal subunit	4.60	1.10×10^{-11}
respiratory chain complex	4.56	4.94×10^{-16}
mitochondrial respirasome	4.53	9.86×10^{-17}

respirasome	4.51	1.78×10^{-18}
endopeptidase complex	4.49	2.89×10^{-13}
U4/U6 x U5 tri-snRNP complex	4.49	8.23×10^{-07}
proteasome complex	4.49	3.18×10^{-12}
U2 snRNP	4.44	1.10×10^{-04}
respiratory chain complex III	4.39	1.34×10^{-03}
mitochondrial respiratory chain complex III	4.39	1.34×10^{-03}
U2-type precatalytic spliceosome	4.37	1.86×10^{-09}
ESCRT III complex	4.35	4.82×10^{-03}
Arp2/3 protein complex	4.35	4.82×10^{-03}
palmitoyltransferase complex	4.35	4.82×10^{-03}
mitochondrial proton-transporting ATP synthase complex	4.35	7.13×10^{-05}
spliceosomal tri-snRNP complex	4.35	1.18×10^{-06}
U1 snRNP	4.32	2.46×10^{-04}
inner mitochondrial membrane protein complex	4.25	3.85×10^{-23}
oligosaccharyltransferase complex	4.21	3.03×10^{-03}
RNA polymerase III complex	4.18	5.45×10^{-04}
proton-transporting ATP synthase complex	4.16	1.01×10^{-04}
mitochondrial protein complex	4.14	8.28×10^{-40}
precatalytic spliceosome	4.13	5.30×10^{-09}
RNA polymerase II, core complex	4.10	1.91×10^{-03}

Table S4. GO-term enrichment of molecular functions.

Go-Term	Fold Enrichment	Raw *p*–Value
peroxiredoxin activity	6.84	8.27×10^{-04}
7S RNA binding	6.84	8.27×10^{-04}
5S rRNA binding	5.59	3.95×10^{-04}
structural constituent of ribosome	5.48	1.26×10^{-42}
peptide disulfide oxidoreductase activity	5.26	2.65×10^{-04}
glutathione binding	5.13	6.14×10^{-04}
threonine-type endopeptidase activity	5.13	7.66×10^{-05}
protein tag	4.88	4.02×10^{-04}
NADH dehydrogenase activity	4.86	2.76×10^{-10}
NADH dehydrogenase (quinone) activity	4.86	2.76×10^{-10}
NADH dehydrogenase (ubiquinone) activity	4.86	2.76×10^{-10}
proton-transporting ATP synthase activity, rotational mechanism	4.78	3.35×10^{-05}
oligopeptide binding	4.73	9.22×10^{-04}
NAD(P)H dehydrogenase (quinone) activity	4.70	2.74×10^{-10}
mRNA 5'-UTR binding	4.37	2.09×10^{-05}
translation initiation factor activity	4.34	1.22×10^{-09}
rRNA binding	4.17	9.54×10^{-11}
phosphatase activator activity	4.02	1.21×10^{-03}
oxidoreductase activity, acting on NAD(P)H, quinone or similar compound as acceptor	3.99	2.03×10^{-09}
glutathione peroxidase activity	3.86	3.07×10^{-04}
2 iron, 2 sulfur cluster binding	3.86	3.07×10^{-04}
translation factor activity, RNA binding	3.64	1.55×10^{-11}
electron transfer activity	3.57	6.24×10^{-13}
oxidoreductase activity, acting on a heme group of donors, oxygen as acceptor	3.54	3.56×10^{-04}
cytochrome-c oxidase activity	3.54	3.56×10^{-04}
proton channel activity	3.54	3.56×10^{-04}
heme-copper terminal oxidase activity	3.54	3.56×10^{-04}
disulfide oxidoreductase activity	3.50	1.51×10^{-05}
NF-κB binding	3.42	1.90×10^{-04}
